# *Akkermansia muciniphila* exacerbates acute radiation–induced intestinal injury by depleting mucin and enhancing inflammation

**DOI:** 10.1093/ismejo/wraf084

**Published:** 2025-04-30

**Authors:** Yafang Wang, Xusheng Wang, Zhenhui Chen, Jihua Zheng, Xiangqiang Liu, Yilin Zheng, Zhihao Zheng, Zi Xu, Yaowei Zhang, Keli Chen, Yuqin Zhang, Lu Yu, Yi Ding

**Affiliations:** Department of Radiation Oncology, Nanfang Hospital, Southern Medical University, Guangzhou, Guangdong, 510515, China; Department of Radiotherapy, General Hospital of Southern Theatre Command, Guangzhou, Guangdong, 510010, China; Department of Radiation Oncology, Nanfang Hospital, Southern Medical University, Guangzhou, Guangdong, 510515, China; Department of Microbiology, Guangdong Provincial Key Laboratory of Tropical Disease Research, School of Public Health, Southern Medical University, Guangzhou, Guangdong, 510515, China; Department of Radiotherapy, General Hospital of Southern Theatre Command, Guangzhou, Guangdong, 510010, China; Department of Gastroenterology, General Hospital of Southern Theatre Command, Guangzhou, Guangdong, 510010, China; Department of Radiation Oncology, Nanfang Hospital, Southern Medical University, Guangzhou, Guangdong, 510515, China; Department of Radiation Oncology, Nanfang Hospital, Southern Medical University, Guangzhou, Guangdong, 510515, China; Department of Radiation Oncology, Nanfang Hospital, Southern Medical University, Guangzhou, Guangdong, 510515, China; Department of Radiation Oncology, Nanfang Hospital, Southern Medical University, Guangzhou, Guangdong, 510515, China; HuiQiao Medical Center, Nanfang Hospital, Southern Medical University, Guangzhou, Guangdong, 510515, China; Department of Radiation Oncology, Nanfang Hospital, Southern Medical University, Guangzhou, Guangdong, 510515, China; Department of Radiation Oncology, Nanfang Hospital, Southern Medical University, Guangzhou, Guangdong, 510515, China; Department of Radiation Oncology, Nanfang Hospital, Southern Medical University, Guangzhou, Guangdong, 510515, China

**Keywords:** Akkermansia muciniphila, acute radiation-induced intestinal injury, mucin, macrophages, goblet cells

## Abstract

Dysbiosis of gut microbiota plays a crucial role in acute radiation-induced intestinal injury. However, studies on the influence of gut microbiota on acute radiation-induced intestinal injury are inconsistent. In this study, we established an acute radiation-induced intestinal injury mouse model and performed fecal microbiota transplantation to explore the role of the gut microbiota in acute radiation-induced intestinal injury. We observed a significant increase in *Akkermansia muciniphila* following irradiation, whereas fecal microbiota transplantation effectively reduced *A. muciniphila* levels. Contrary to expectations, *A. muciniphila* supplementation increased acute radiation-induced intestinal injury and mortality. Mechanistically, postradiation *A. muciniphila* upregulates mucin metabolism genes and consumes mucin, thinning the mucosal barrier and promoting the adhesion and translocation of potential pathogens to epithelial cells, thus exacerbating acute radiation-induced intestinal injury. This enables *A. muciniphila* to use mucin as an energy source. Additionally, *A. muciniphila* increases the inflammatory macrophage changes and secretion of inflammatory cytokines, leading to a decrease in epithelial stem cell density and inhibition of goblet cell differentiation, further exacerbating acute radiation-induced intestinal injury. Our findings suggest that in certain intestinal environments, the addition of *A. muciniphila* may worsen radiation-induced intestinal damage; thus, alternative approaches to reverse the dysbiosis associated with radiotherapy should be explored.

## Graphical Abstract

**Graphical Abstract:**
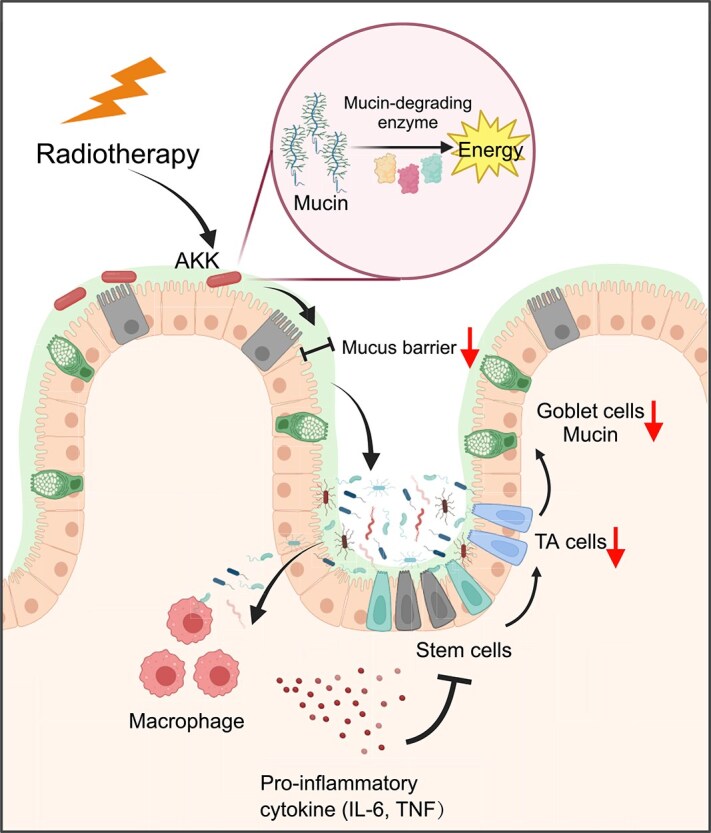

## Introduction

Radiotherapy is a common treatment for abdominal and pelvic tumors; however, up to 75%–81% of patients undergoing this therapy experience acute radiation–induced intestinal injury (ARIII) [1, 2]. This condition not only poses a major limitation to improving tumor prognosis but also severely affects patients’ quality of life and can even lead to death [3–5]. Currently, there are no reliable tools for predicting radiation injuries or strategies for their prevention or treatment.

Increasing evidence has confirmed that dysbiosis of the gut microbiota plays a critical role in ARIII [6–8]. However, data on the mechanism of the influence of gut microbiota on radiation-induced intestinal damage are contradictory. One clinical study showed that administering specific bacterial species (mainly *Lactobacillus*) as probiotics could reduce intestinal damage postradiation [9], but other studies indicated a lack of significant protective effects [10]. Some studies have suggested that changes in probiotics after radiotherapy can exacerbate intestinal damage. A previous study showed that radiation induces dysbiosis, which transmits radiation and inflammatory susceptibility and provides evidence that microbial-induced radiation tissue damage is at least in part mediated by IL-1β [11]. Although probiotics are generally safe, they should be used cautiously in patients with radiation-induced intestinal injury because the bacterial strains, quantities, and host responses may vary.

Recently, the regulatory role of ARIII on immune cells has become increasingly recognized [8]. High-dose radiation exposure can cause immune function decline or suppression [12]. Macrophages, one of the largest groups of immune cells in the body, are major participants in establishing and maintaining intestinal homeostasis [13–15] and are new targets for radiation injury treatment [16]. When radiation damages intestinal tissues, pathogenic bacteria interact with intestinal epithelial cells, exacerbating enteritis by affecting the function and status of macrophages [13]. Intestinal stem cells located at the bottom of crypts are crucial for repairing intestinal damage [17]. They can differentiate into rapidly proliferating transit-amplifying (TA) cells, which then differentiate into intestinal, goblet, and endocrine cells, thus playing a vital role in maintaining intestinal homeostasis [18, 19]. Studies have indicated that the supernatant of *Lactobacillus johnsonii* alleviates colitis in mice by modulating macrophages and remodeling the gut microbiota [20]. Moreover, these microorganisms also affect macrophages. For example, *A. muciniphila* induces a trained immune phenotype in macrophages, enhancing bacterial intracellular survival and reducing inflammation [21]. Similarly, *Lactiplantibacillus plantarum* induces long-term memory-like immune responses in macrophages, improving bacterial survival and reducing inflammation [22]. Therefore, these studies highlight the importance of microorganisms in modulating macrophage function and their potential therapeutic applications in immune modulation.

*Akkermansia muciniphila* (AKK) has received considerable attention owing to its role in regulating immune responses and enhancing intestinal barrier function. Studies have confirmed that short-chain fatty acids produced by AKK can activate the G-protein-coupled receptor GPR4, thereby regulating Foxp3+ Tregs and alleviating intestinal inflammation [23]. They also mitigate acute colitis induced by dextran sulfate sodium by activating the NLRP3 inflammasome [24]. However, the role of AKK in maintaining host intestinal health remains incompletely understood. Evidence suggests that excessive enrichment of AKK in mice under specific intestinal conditions may exacerbate intestinal inflammation, particularly in the context of epithelial barrier damage [25, 26]. Moreover, in colorectal cancer (CRC) mouse models receiving fecal microbiota transplants from patients with colorectal cancer, the abundance of AKK has been positively associated with increased tumor burden [27]. Additionally, gavage of AKK to intestine-specific *Apc* mutant mice has been shown to promote colorectal cancer progression by increasing tumor multiplicity [28].

In the present study, we observed a significant increase in AKK levels in the ARIII model of male C57BL/6 J mice. Following fecal microbiota transplantation (FMT) from healthy donors, AKK abundance decreased once again, which piqued our interest. Our data indicated that postradiation, a high abundance of AKK in the gut heavily consumes mucin as an energy source to combat radiation-induced stressors. Thinning of the mucosal barrier, primarily composed of mucin, exacerbates the adhesion and translocation of pathogenic bacteria to intestinal epithelial cells, thereby triggering macrophage-dominated immune activation. Inflammatory cytokine activation suppresses the intestinal stem cell–TA cell–goblet cell axis. Reduced numbers of goblet cells lead to decreased mucin secretion, fostering a vicious cycle that enhances pathogen invasion, thereby aggravating intestinal damage. This study highlights the adaptive behavior of AKK under extreme conditions (high-dose radiation), prompting cautious consideration of whether AKK may serve as a universal probiotic.

## Materials and methods

### Patient and sample collection

Baseline fresh fecal samples were collected from patients aged 18–75 years with locally advanced rectal adenocarcinoma who exhibited mismatch repair proficiency or microsatellite stability, which was confirmed by genetic testing prior to the initiation of neoadjuvant radiochemotherapy. Acute radiochemotherapy–induced intestinal injury was assessed during treatment based on the Common Terminology Criteria for Adverse Events guidelines. Patient specimens and data were collected in compliance with the guidelines of the Ethics Committee of Nanfang Hospital, Southern Medical University (approval number: NFEC-2023-219). Written informed consent was obtained from all patients prior to fecal sample collection, and patients retained the right to withdraw their consent at any time during the study.

### Animal model

All animal experiments were approved by the Institutional Animal Care and Use Committee of Nanfang Hospital, Southern Medical University. Six- to eight-week-old male C57BL/6 J mice (18–20 g) were purchased from Zhiyuan Biotechnology and Pharmaceutical Technology Company (Guangzhou, China) and raised under specific pathogen-free conditions, with free access to food and drinking water. Before the experiment, the mice were allowed to adapt to the experimental environment for 1 week. The mice were randomly divided into groups without blinding.

The radiochemotherapy (RCT) model was established by treatment with a total abdominal irradiation dose of 6 Gy (Varian Clinac 23EX Linear Accelerator; Varian Medical Systems, USA) and three intraperitoneal injections of fluorouracil (50 mg/kg). The irradiation (IR) model was established by treatment with a total abdominal irradiation dose of 14 Gy (Varian Clinac 23EX Linear Accelerator; Varian Medical Systems, USA). For the FMT treatment, fresh feces from the same mice were collected when they were in a healthy state before irradiation and stored at −80°C. Fecal samples (200 mg) were resuspended in 1 ml of sterile anoxic phosphate-buffered saline (PBS) and centrifuged at 800 × *g* for 5 min. The supernatant was then passed through a 70 μm filter to remove large particulates. The mice were intragastrically administered 0.2 ml fresh fecal solution once daily for 3 days. For the treatment group, mice treated with AKK received 1 × 10^9^ CFU/200 μl/day intragastrically for 7 days. Mice treated with antibiotics (ABX) were orally administered an ABX cocktail of ampicillin (1 mg/ml, Solarbio), neomycin (1 mg/ml, Solarbio), metronidazole (1 mg/ml, Solarbio), and vancomycin (0.5 mg/ml, Sigma-Aldrich) for 4 days. Mice treated with the M1 inhibitor were intraperitoneally injected with 10 mg/ml lupeol (MedChemExpress) for 4 days. The mice in each group were euthanized on day 4 postirradiation to collect blood, intestinal contents, feces, and intestinal tissues for further study. Mice in each group were monitored for survival until day 30 postirradiation. The specific animal model experimental design and the number of mice are detailed in the figure and figure legend.

### Bacterial culture and *in vitro* gut microbe culture model

*Akkermansia muciniphila* Muc was purchased from the ATCC (BAA-835) and cultured in basal liquid brain heart infusion (BHI) medium (Huaikai Bio-Technology, China) supplemented with 0.2% mucin (Yuanye Bio-Technology, China) for 48 h under an anoxic incubator (Shanghai Chuanhong, YQX-II, Guangzhou Yilong Technology Co., Ltd., China, with 5% H_2_, 10% CO_2_, and 85% N_2_) at 37°C. Among these, live *Akkermansia* refers to untreated bacteria that retain their metabolic activity and actively interact with the gut microbiota. Pasteurized *Akkermansia* was prepared using heat treatment at 70°C for 30 min, preserving certain biological effects but eliminating full metabolic function. Lastly, heat-inactivated *Akkermansia* was treated at 100°C for 5 min, resulting in minimal biological activity owing to exposure to high temperatures. For different mucin incubation experiments, 1 × 10^8^ CFU of AKK were inoculated into BHI medium containing 0.5%, 1%, or 2% mucin in 96-well plates and exposed to 4 or 8 Gy irradiation. After 12 or 24 h of cultivation, the OD_600_ was used to detect the bacterial content.

An *in vitro* gut microbial culture model was established as previously reported [29]. Briefly, 100 mg of fresh fecal samples from mice were collected and dropped into tubes containing 0.5 ml sterile PBS prereduced with 0.1% (w/v) L-cysteine hydrochloride. The samples were immediately transferred to an anoxic workstation, 5% H_2_, 10% CO_2_, and 85% N_2_) at 37°C. Before homogenization using a vortex mixer, the tube was uncapped for a few seconds to enable gas exchange and oxygen removal. Sample homogenates were filtered using sterile gauze and immediately inoculated into each medium for static culture at a final inoculum concentration of 2% (w/v). Individual microbiomes were cultured in 1 ml of MiPro medium. The MiPro medium contained 2.0 g/l peptone water, 2.0 g/l yeast extract, 0.5 g/l L-cysteine hydrochloride, 2 ml/l Tween 80, 5 mg/l hemin, 10 μl/l vitamin K1, 1.0 g/l NaCl, 0.4 g/l K_2_HPO_4_, 0.4 g/l KH_2_PO_4_, 0.1 g/l MgSO_4_·7H2O, 0.1 g/l CaCl_2_·2H2O, 4.0 g/l NaHCO_3_, 4.0 g/l porcine gastric mucin, and 0.5 g/l bile salts (Sigma-Aldrich).

### Hematoxylin and eosin staining

The mouse intestinal tissues were isolated 4 days after radiation therapy, rinsed with ice-cold PBS, and immediately transferred to 10% neutral-buffered formalin. The intestinal tissues were processed by gradient dehydration, embedded, and sectioned into 5 μm slices for hematoxylin and eosin (H&E) staining. H&E staining was performed according to the manufacturer’s instructions (Solarbio, Beijing, China). An established scoring criterion was used to conduct the pathological injury assessment by two independent pathologists blinded to the study. The criteria were as follows: 0 (normal), no damage; 1 (mild), slight submucosal and/or lamina propria separation; 2 (moderate), moderate separation of the submucosa and/or lamina propria and/or edema in the submucosal and muscular layers; 3 (severe), severe separation of the submucosa and/or lamina propria and/or severe edema in the submucosa and muscular layers, along with villous sloughing; and 4 (necrosis), loss of villi and necrosis.

### Alcian blue–periodic acid–Schiff staining and immunohistochemical staining

For goblet cell detection, Alcian blue–periodic acid-Schiff (AB-PAS) staining was performed using an AB-PAS Stain Kit (Solarbio, Beijing, China), according to the manufacturer’s recommendations. Images were captured using a microscope (DP22, Olympus). The total number of goblet cells in crypts from five different fields was counted.

Immunohistochemical (IHC) staining of paraffin-embedded mouse intestinal tissue sections was performed according to standard protocols. Sections were deparaffinized, rehydrated, subjected to antigen retrieval, and blocked with 3% hydrogen peroxide and goat serum, followed by overnight incubation with primary antibody (rabbit anti-Muc2 antibody, 1:2000, ab272692; Abcam) at 4°C. The next day, the sections were incubated at room temperature for 30 min to rewarm, followed by incubation with the secondary antibody for 1 h at room temperature, and 3,3′-diaminobenzidine (DAB) staining was performed using an IHC assay kit (Maixin, China). Counterstaining was performed using hematoxylin for 2 min. Images were captured using a microscope. Staining intensity was independently evaluated by two senior pathologists. IHC scores were calculated based on the positive reaction area and staining intensity. Each group was quantified on the basis of at least five different views.

### Multiplex immunochemical analysis

Formalin-fixed, paraffin-embedded tissue sections (5 μm) were deparaffinized and rehydrated through graded alcohol solutions to water. Antigen retrieval was performed using a citrate buffer (pH 6.0) in a pressure cooker at 95°C for 20 min. Endogenous peroxidases were blocked with 3% hydrogen peroxide for 10 min at room temperature, followed by blocking with 5% bovine serum albumin in PBS for 1 h to reduce nonspecific binding. The sections were then incubated with the primary antibodies for 1 h at room temperature. After washing three times with Tris-buffered saline with 0.1% Tween 20 (TBST), the sections were incubated for 10 min with HRP-conjugated goat anti-rabbit IgG H&L (1:1000, ab6721; Abcam). After washing three times with TBST, the sections were incubated for 10 min with a tyramide signal amplification reagent. This procedure was repeated until all primary antibodies (rabbit anti-Olfm4 antibody, 1:400, 39141 T, CST; rabbit anti-Pcna antibody, 1:800, 13110 T, CST; rabbit anti-Muc2 antibody, 1:500, ab272692, Abcam) were incubated. Finally, sections were counterstained with 4′,6-diamidino-2-phenylindole (DAPI) and observed under a fluorescence microscope. Fluorescence intensity was quantitatively analyzed using ImageJ software.

### Metagenomic sequencing

Fecal samples from mice were collected and immediately frozen at −80°C until DNA extraction. Genomic DNA was extracted using a QIAamp PowerFecal DNA Kit (Qiagen), according to the manufacturer’s protocol. DNA concentration and purity were assessed using a Qubit 3.0 Fluorometer and a NanoDrop ND-1000 spectrophotometer, respectively. Metagenomic libraries were prepared using the Nextera XT DNA Library Preparation Kit (Illumina), which involves enzymatic tagmentation for DNA fragmentation, followed by the addition of dual indices and adapters for sequencing. Library quality and size were validated using an Agilent 2100 Bioanalyzer. Sequencing was conducted on an NovaSeq 6000 platform (Illumina), producing 150 bp paired-end reads. Raw reads were subjected to quality control using Trimmomatic (v0.39) to trim adapters and filter out low-quality reads (Phred score < 20). High-quality reads were aligned to the human reference genome (GRCh38) using Bowtie 2 (v2.3.5.1) to exclude human-derived sequences. Nonhuman reads were assembled into contigs using MEGAHIT (v1.2.9), and open reading frames were predicted using Prodigal (v2.6.3). DIAMOND (v0.9.24) was used for functional annotation against the National Center for Biotechnology Information nonredundant protein database to identify microbial genes linked to metabolic pathways. Taxonomic profiling was performed using Kraken2 (v2.1.1) based on *k*-mer alignment against a comprehensive microbial database. Statistical analyses were performed in R (v4.0.3) using the vegan and edgeR packages for community ecology and differential abundance testing, respectively.

### 16S ribosomal RNA (rRNA) gene sequencing

Fecal samples were collected and stored at −80°C until processing. RNA was extracted using a Fecal RNA Extraction Kit (Solarbio, China), according to the manufacturer’s instructions. Briefly, 0.25 g of fecal material was homogenized in lysis buffer and RNA was isolated using phenol–chloroform extraction, followed by silica column purification.

Sequencing data quality was assessed using Trimmomatic (version 0.33) to remove low-quality reads and to trim adapters. Primer sequences were removed using Cutadapt (version 1.9.1) with a maximum mismatch rate of 20% and a minimum overlap of 80%. Paired-end reads were assembled using USEARCH (version 10), which required a minimum overlap length of 10 bp and a similarity threshold of 90%. The chimeric sequences were identified and removed using UCHIME (version 8.1). High-quality sequences were clustered into operational taxonomic units (OTUs) using USEARCH at a 97% similarity threshold. Sequences representing <0.005% of the total reads were discarded. For a higher taxonomic resolution, denoising and amplicon sequence variant (ASV) generation were performed using the DADA2 algorithm in QIIME2 (version 2020.6) by applying a filtering threshold of 0.005% of the total reads. Taxonomic classification of OTUs and ASVs was conducted using the SILVA Comprehensive Ribosomal RNA Database (SILVA database, release 138) with the classify-sklearn algorithm in QIIME2, employing a pretrained Naive Bayes classifier. Sequences that did not align well were classified using a classify-consensus-blast with a minimum similarity of 90% and a coverage of 90%. Alpha diversity indices, including the Shannon Index, were calculated using QIIME2. Beta diversity analyses, including principal component analysis (PCA), were performed and visualized using R software, based on Bray–Curtis dissimilarity. A ternary plot analysis was used to compare the species composition of three or three sets of samples. The ternary plot was created using the Plotly library in Python. Each point represents a species, and the position of the point indicates its relative abundance across the three sets of samples. Points closer to the vertices represent species with higher abundance in the corresponding group, whereas points closer to the center indicate a more balanced abundance across the three groups. BugBase is a method for predicting functional pathways within complex microbiomes, which provides biological-level coverage and biologically interpretable phenotypes. BugBase normalizes OTUs based on the predicted 16S rRNA gene copy number and then uses the provided precomputed files to predict microbial phenotypes [30].

### Fluorescence *in situ* hybridization

Fluorescence *in situ* hybridization (FISH) was performed using established protocols [31] to assess AKK abundance and gut microbiota adherence to jejunum epithelial cells. Paraffin-embedded tissue sections (5 μm) were deparaffinized and rehydrated through sequential washes in xylene and ethanol. Antigen retrieval involved boiling the sections in 1× retrieval solution. Proteinase K digestion was performed at 37°C for 20 min. The sections were then incubated with preheated hybridization solution (40°C) in a humidified chamber for 30 min for prehybridization. For hybridization, ~60 μl of preheated probe mix was applied and the sections were incubated overnight at 40°C in a humidified chamber. Posthybridization, the sections were sequentially washed with preheated 2× saline sodium citrate (SSC), 1 × SSC, 0.5 × SSC, and 0.1 × SSC at 40°C (5 min each), with adjustments made for nonspecific binding. Finally, the sections were counterstained with DAPI and observed under a fluorescence microscope. The probe sequences used were eub338 (5′CY3-GCTGCCTCCCGTAGGAGT-3′) and AKK (5′Alexa 488-CCTTGCGGTTGGCTTCAGAT-3′). Fluorescence intensity was quantitatively analyzed using ImageJ software.

### Enteroids

A 10 cm segment of the small intestine of 5-week-old C57BL/6 J mice was excised immediately and rinsed with precooled (4°C) PBS. Adipose tissues and the mesentery were meticulously removed from the intestine, and the intestinal contents were cleared with prechilled PBS at 4°C. The small intestine was then segmented into 2 mm pieces, which were gently pipetted six times with PBS to further separate the tissue. The tissue fragments were allowed to settle, and the supernatant was discarded. The precipitates were treated with PBS containing ethylenediaminetetraacetic acid (EDTA) and incubated for 30 min at 4°C on a shaker. After incubation, the tissue was pipetted six times with PBS to disperse the crypts. Between 200 and 500 crypts/well were then embedded in Matrigel (Corning) and cultured in IntestiCult Organoid Growth Medium (Stemcell). The medium was refreshed every 2–3 days. Two days prior to experimentation, the organoids were transferred into organoid culture medium without antibiotics. Immediately following bacterial addition, organoids were subjected to radiation treatment (RT). Bacteria were directly added to the culture medium and co-incubated with the organoids for 8 h (4 h post-RT). The domes were then washed five times with PBS containing antibiotics to remove residual bacteria, followed by replacing the medium with standard organoid culture medium. The organoids were subsequently cultured for an additional 24 h. Enteroids were treated with 4 Gy of irradiation and/or 1 × 10^6^ CFU/10 μl of live AKK, 1 × 10^6^ CFU/10 μl of inactivated AKK, 10 μl of *in vitro* gut microbe culture model medium, 1 × 10^4^ unpolarized macrophages or classically activated macrophages, and/or 10 mM of M1 inhibitor. Images were captured using a microscope.

### Cell culture

The Raw264.7 cell line was generously provided by the Department of Pathology of Nanfang Hospital and cultured in Dulbecco’s modified Eagle’s medium supplemented with 10% fetal bovine serum at 37°C under 5% CO_2_. M1 polarization was induced by treating the cells with 100 ng/ml lipopolysaccharide (MedChemExpress) for 48 h.

### RNA sequencing and gene set variation analysis

Total RNA was extracted from the jejunum using TRIzol reagent (Takara, Japan). Approximately 60 mg of tissue was powdered in liquid nitrogen, homogenized, and centrifuged to isolate RNA. Chloroform/isoamyl alcohol was used to extract the RNA, which was then precipitated using isopropyl alcohol. Purified RNA was quantified, and its quality was assessed using a NanoDrop and Agilent 2100 (Thermo Fisher). Oligo (dT)-attached beads were used to isolate the messenger RNA (mRNA), which was fragmented and reverse-transcribed into complementary DNA (cDNA). The cDNA was purified, end-repaired, and ligated with adapters for polymerase chain reaction (PCR) amplification. The quality and quantity of the cDNA library were checked using an Agilent 2100 and quantitative polymerase chain reaction (qPCR). The library was duplex-specific nuclease-treated, flow-cell-amplified, and single-end-sequenced on a NovaSeq 6000 (Illumina) to obtain high-quality sequencing data (Tsingke-Beijing).

Marker genes for immune and intestinal epithelial cells from the gut were downloaded and compiled using CellMarker 2.0 (http://bio-bigdata.hrbmu.edu.cn/CellMarker/index.html). The gene set variation analysis (GSVA) R package was used for immune cell infiltration analysis and subtype characterization of the intestinal epithelial cells. The Wilcoxon rank-sum test was used for the statistical analysis of cell abundance. Data visualization was performed using the ImageGP platform [32].

Real-time quantitative reverse transcription–polymerase chain reaction (qRT-PCR): Samples consisting of 100 mg of fresh feces from patients with rectal cancer, AKK, or jejunum were homogenized in 1 ml TRIzol reagent (Takara, Japan). RNA was extracted using the chloroform–isopropanol method. RNA from bacteria or tissues was reverse-transcribed into cDNA using Evo M-MLV RT Master Mix (AG, China), according to the manufacturer’s instructions. Subsequently, mRNA expression was analyzed using a SYBR Green Premix Pro Taq HS qPCR Kit (Rox Plus) (AG, China) with a LightCycler 96 detection system (Roche). The16S rRNA gene or β-actin was used for normalization. The primer sequences used are listed in Table S1.

### Enzyme-linked immunosorbent assay (ELISA)

Blood samples were collected from the mice and centrifuged at 300 × *g* for 20 min. The supernatants were collected for ELISA. Jejunum tissues in each group were ground in 400 μl of PBS per 0.4 g, followed by centrifugation for 10 min at 4000 × *g* and 4°C. IL-6, TNF, and TGF-β levels in the blood and tissues were measured using ELISA kits (Neobioscience, China) according to the manufacturer’s instructions.

### Flow cytometry

On Day 4 postirradiation, mice from each group were euthanized and small intestinal tissues were collected. The tissues were washed with PBS to remove intestinal contents. Next, the tissues were incubated in PBS prewarmed to 37°C and supplemented with 5 mM DTT and 5 mM EDTA, followed by horizontal shaking at 220 rpm and 37°C for 30 min. After vortexing for 30 s, the buffer was discarded. The tissues were rinsed twice with PBS, transferred to EP tubes, and cut into 1 mm^2^ pieces. These pieces were then incubated in roswell park memorial institute (RPMI) medium containing 2 mg/ml collagenase IV and subjected to horizontal shaking at 220 rpm and 37°C for 1 h. Next, the cell suspension was filtered through a 70 μm nylon mesh and centrifuged at 500 × *g* for 5 min at 4°C. The supernatant was then discarded, and the cell pellet was resuspended in fresh fluorescence-activated cell sorting (FACS) buffer, followed by washing with PBS twice. Cells were incubated with an Fc receptor blocker (abs9477; Absin, Shanghai, China) at 4°C for 15 min and washed again to obtain the cell pellet. Subsequently, cells were stained with macrophage-associated surface monoclonal antibodies, including APC-Cy7-conjugated CD45 (557659; BD Pharmingen, San Diego, CA, USA), fluorescein isothiocyanate (FITC)-conjugated CD11b (557396; BD Pharmingen, San Diego, CA, USA), reticulocyte binding (RB) 705-conjugated F4/80 (570288; BD Pharmingen, San Diego, CA, USA), and phycoerythrin (PE)-conjugated CD86 (553692; BD Pharmingen, San Diego, CA, USA), and incubated at 4°C for 30 min. The single-cell suspension was filtered through a nylon mesh and analyzed using a flow cytometer. Pro-inflammatory macrophages were gated as CD45^+^F4/80^+^CD11b^+^CD86^+^ cells.

### Statistical analysis

All data are expressed as mean ± standard deviation, and statistical significance was set at *P* ≤ .05. Statistical differences between the experimental groups were analyzed using one-way ANOVA and Student’s *t*-test. Differences in survival between the groups were assessed using Kaplan–Meier curves and the log-rank test. All statistical analyses were performed using SPSS (version 22.0; IBM, NY, USA). All experiments were performed in triplicate.

## Results

### Abnormally high abundance of *A. muciniphila* in the gut following radiotherapy

**Figure 1:**
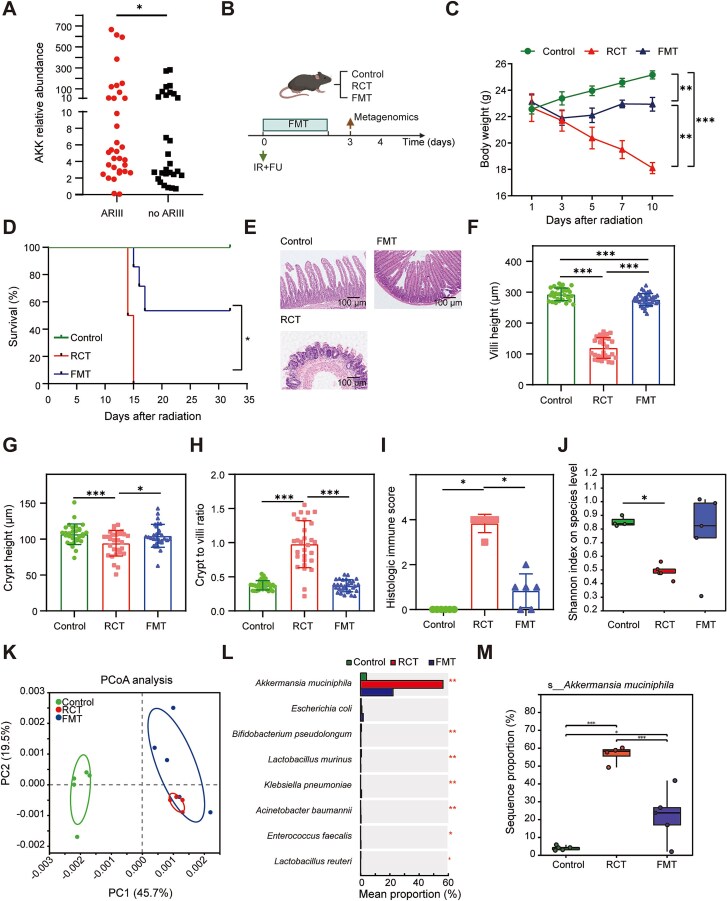
Abnormally high abundance of *A. muciniphila* in the gut following radiotherapy. (A) Relative abundance of AKK in the baseline feces of rectal cancer patients before cancer treatments. (B) Timeline and experimental design of the experiment (control *n* = 6, IR *n* = 6, IR + FMT *n* = 7). Metagenomics analysis was performed on Day 4. (C) Body weight was monitored after postirradiation. (D) Survival analysis of mice in the three groups was conducted over 35 days postirradiation. (E) Representative images of HE stained of jejunum among the three groups. (F–I) Bar graph showing the crypt height (F), villus height (G), crypt-to-villus ratio (H), and histologic immune scores (I) across the three groups. (J) Shannon Index of gut microbiome was measured in the three groups. (K) PCA of gut microbiome was measured in the three groups. (L) The abundance of key bacterial taxa. (M) Proportion of *A. muciniphila* across the three groups. ^*^Significance levels: ^*^P < .05, ^*^^*^P < .01, ^*^^*^^*^P < .001.

By collecting baseline fecal samples from patients with locally advanced rectal cancer prior to neoadjuvant chemoradiotherapy and quantifying the abundance of AKK using qRT-PCR, our results demonstrated that patients who developed ARIII after radiotherapy exhibited a significantly higher abundance of AKK in their gut microbiota compared with those who did not (Fig. 1A). To simulate clinical situations, we established a mouse model and employed FMT to assess the impact of the gut microbiota on ARIII (Fig. 1B). Our findings demonstrated significant weight loss and increased mortality in mice postirradiation; mice receiving FMT exhibited gradual weight recovery and markedly improved survival (Fig. 1C and D). Histological examination using H&E staining revealed extensive epithelial damage, evident edema and congestion, increased inflammatory cell infiltration, significantly reduced crypt and villus height, diminished crypt-to-villus ratio, and elevated inflammation scores in mice subjected to irradiation (Fig. 1E–I). Conversely, FMT administration led to notable improvements in the epithelial integrity, crypt–villus architecture, and inflammatory scores. These results confirmed the substantial ameliorative effects of FMT on intestinal injury.

Metagenomic sequencing was performed to delineate the specific microbial influence on intestinal injury. Ace index analysis indicated a marked reduction in the alpha diversity of the gut microbiota in irradiated mice, which was partially restored by FMT treatment; however, this change was not statistically significant (Fig. 1J). Principal Coordinates Analysis (PCoA) revealed distinct compositional differences in the gut microbiota among the three groups, highlighting the ability of FMT to alter the gut microbiota composition (Fig. 1K). Species-level differential analyses revealed significant changes in several taxa, including *Bifidobacterium pseudolongum* and *Lactobacillus murinus*. In particular, the pronounced alteration of AKK garnered attention (Fig. 1L). After irradiation, AKK abundance significantly increased in the gut, whereas FMT led to a marked reduction (Fig. 1M). Collectively, these findings suggest that AKK plays a pivotal role in the onset and progression of intestinal injury after cancer therapy.

### Mucin uptake by *A. muciniphila* as a self-protective measure postradiation exacerbates acute radiation–induced intestinal injury

**Figure 2:**
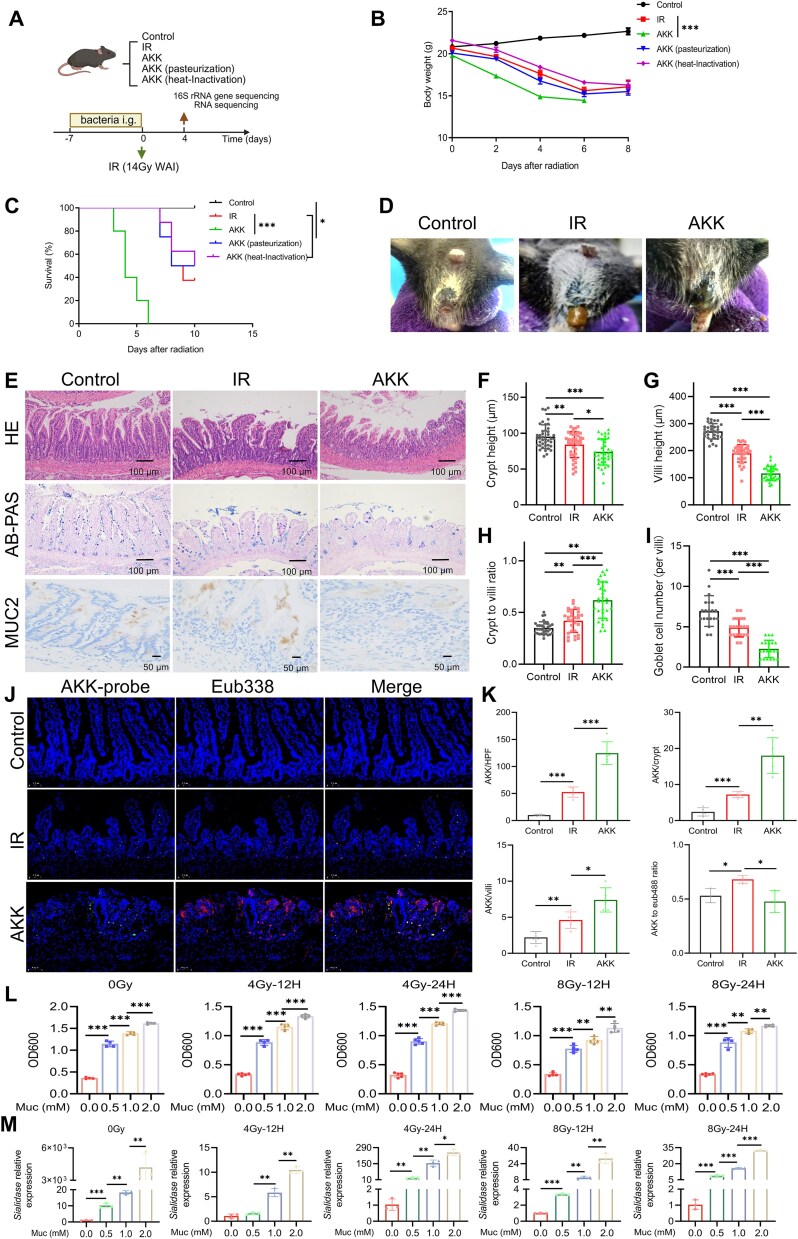
Impact of *A. muciniphila* on radiation-induced intestinal injury and gut microbiota composition. (A) Experimental design of the experiment (control *n* = 5, IR *n* = 8, AKK *n* = 8, AKK-pasteurization *n* = 8, AKK-heat-inactivation *n* = 8). (B) Body weight changes of mice. (C) Survival analysis of mice after postirradiation. (D) Representative picture of fecal consistency changes of mice at Day 4 after irradiation. (E) Histopathological analysis of jejunum sections with HE staining, AB-PAS staining, and IHC staining of Muc2. (F–I) Quantification of crypt height (F), villus height (G), crypt-to-villus ratio (H), and number of goblet cells (I) across the different treatment groups. (J) FISH staining of AKK colonization in the jejunum. (K) Quantification of AKK in the high power field (HPF), crypt, villus, and fluorescence intensity ratio of AKK to Eub338. Bacteria, red; DNA, blue; AKK, green. (L) The growth rate of *A. muciniphila* cultured in medium with different concentrations of mucin. (M) The relative mRNA expression levels of mucin-utilizing genes were examined. ^*^Significance levels: ^*^P < .05, ^*^^*^P < .01, ^*^^*^^*^P < .001.

To ascertain the pivotal role of AKK in ARIII, we treated the model mice with live, heat-inactivated, or pasteurized AKK (Fig. 2A). Mice in the AKK group exhibited significant weight loss by Day 4 (Fig. 2B), increased mortality (Fig. 2C), and notable changes in fecal consistency (Fig. 2D), all of which were statistically distinct from those in the IR group. Conversely, no statistical differences in weight or survival rate were observed between the AKK-pasteurized, AKK-heated, and IR groups (Fig. 2B and C). Analysis of fecal samples revealed markedly elevated AKK levels in the AKK-treated group (Fig. S1A). Necropsy of mice euthanized on Day 4 postirradiation showed shortened colonic lengths in the AKK group compared to those in the IR group (Fig. S1B), whereas morphological changes in spleen size and weight were not statistically significant (Fig. S1C). These findings highlight the detrimental effects of AKK.

To determine whether AKK exacerbated intestinal damage, leading to diarrhea and increased mortality, we performed histopathological staining of various intestinal segments (Figs 2E and S1D). The results indicated exacerbated pathological lesions in all intestinal segments of mice in the AKK group compared to those in the IR group, with the most pronounced damage observed in the jejunum, accompanied by further reductions in villus height, crypt depth, and crypt-to-villus ratio (Fig. 2F–H). H&E staining revealed a decrease in the number of goblet cells in the villi of mice in the AKK group, which was corroborated by AB-PAS staining (Fig. 2E and I). Immunofluorescence staining confirmed a significant reduction in Muc2, a critical product of goblet cells, in the AKK group (Fig. 2E).

To ascertain the localization and functional implications of AKK in exacerbating intestinal damage, FISH analysis demonstrated increased AKK colonization in the intestinal villi, correlating with decreased villus height, crypt depth, goblet cell count, mucin expression, and increased immune scores (Fig. 2E–K and S1E). To determine whether AKK utilizes mucin as a crucial energy source postradiation and enhances mucin uptake, we conducted *in vitro* AKK culture experiments using varying mucin concentrations. The results indicated increased mucin concentrations with higher AKK survival rates postirradiation, highlighting the dependence of AKK on mucin (Fig. 2L). PCR amplification analysis further confirmed the significant upregulation of mucin-utilizing genes, such as *sialidase*, *aga*, *ana*, and *β-galactosidases* in AKK postirradiation (Figs 2M and S1F).

### *A. muciniphila* increases the abundance of potential pathogens in the jejunum of mice with acute radiation–induced intestinal injury

**Figure 3:**
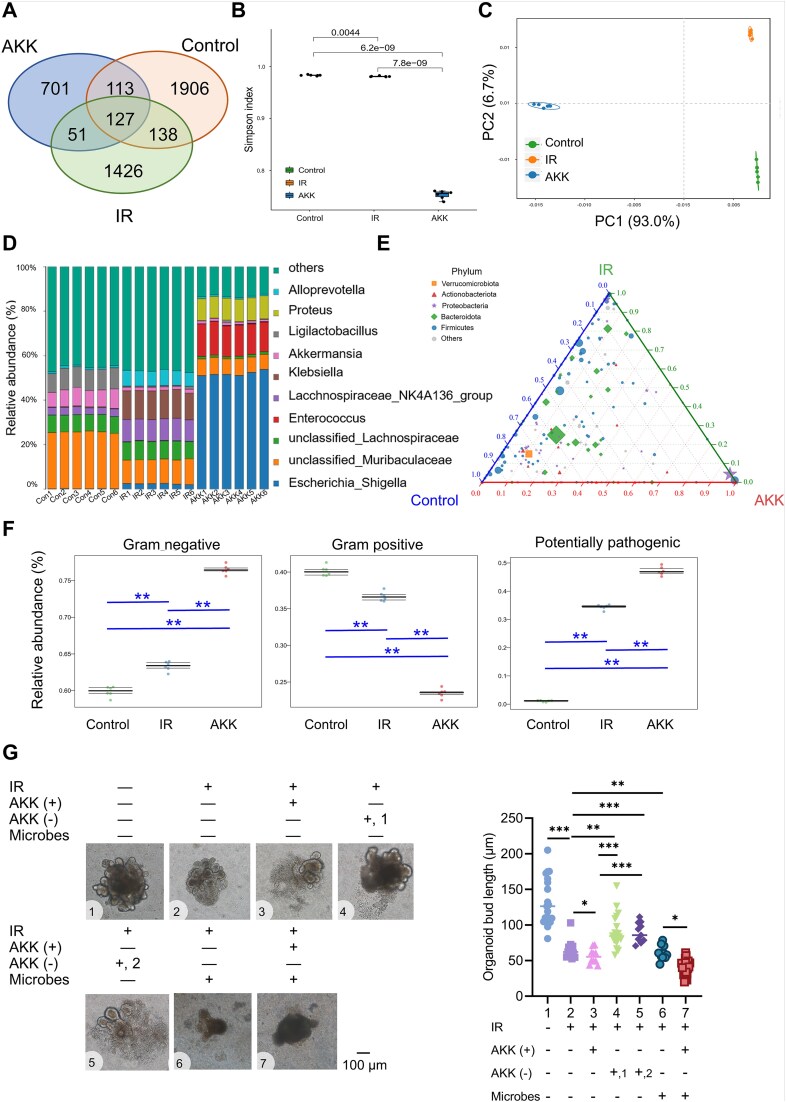
*A. muciniphila* increases the abundance of potential pathogens in the intestines of mice with RIII. (A) Venn diagram showing unique and shared operational taxonomic units (OTUs) among three groups. (B) Shannon Index of fecal microbiome was measured in the three groups. (C) PCA of fecal microbiome was measured in the three groups. (D) The relative abundance of dominant bacterial strains at the species level. (E) Ternary plot depicting the distribution of bacterial phyla. (F) The relative abundances of Gram-negative, Gram-positive, and potentially pathogenic bacteria were analyzed by BugBase phenotype prediction. (G) Representative images of enteroids cultured under various conditions: IR alone, with/without live AKK (+), with/without inactivated AKK (−) (1, heat-inactivated, 2, pasteurized-inactivated), and with/without *in vitro* gut microbe culture. ^*^Significance levels: ^*^P < .05, ^*^^*^P < .01, ^*^^*^^*^P < .001.

In the preceding section, we observed a potential association between localized AKK aggregation and increased bacterial adherence to intestinal epithelial cells. To confirm whether this phenomenon was induced by AKK, we collected small intestine contents from mice on Day 4 postirradiation from the control, IR, and AKK groups for qPCR and 16S rRNA gene sequencing. The qRT-PCR results revealed differential colonization levels of AKK across the intestinal contents, with significantly higher AKK abundance observed in the jejunum and ileum of mice in the AKK group (Fig. S2A). The results of sequencing indicated significant differences in the intestinal bacterial species and composition among the control, IR, and AKK groups (Fig. 3A). Alpha diversity analysis using the Shannon Index revealed markedly reduced diversity in the AKK group compared to that in the IR group (Fig. 3B). PCA and taxonomic composition analyses demonstrated distinct differences in gut microbiota composition among the three groups (Fig. 3C and D). Ternary plot analysis showed an altered distribution of intestinal microbiota in all groups, with an increased abundance of *Actinobacteriota* and *Proteobacteria* in the AKK group compared to that in the IR group, in addition to increases in *Bacteroidota* levels (Fig. 3E). Bugbase phenotype prediction highlighted a gradual increase in Gram-negative bacteria, potentially pathogenic, facultatively anaerobic, and stress-tolerant species in the control, IR, and AKK groups, whereas Gram-positive bacteria showed a decreasing trend (Figs 3F and S2B). Further *in vitro* experiments using enteroids explored whether the harmful effects of AKK depend on other bacteria. The results indicated a slight increase in damaged cells when live AKK bacteria were added postirradiation, with no additional increase observed when heat-inactivated or pasteurized-inactivated AKK bacteria were added. However, severe damage to enteroids occurred when both live AKK bacteria and total gut microbiota were simultaneously added to the culture, suggesting that AKK exacerbation of ARIII depends on other intestinal bacteria, particularly pathogenic species (Fig. 3G).

**Figure 4:**
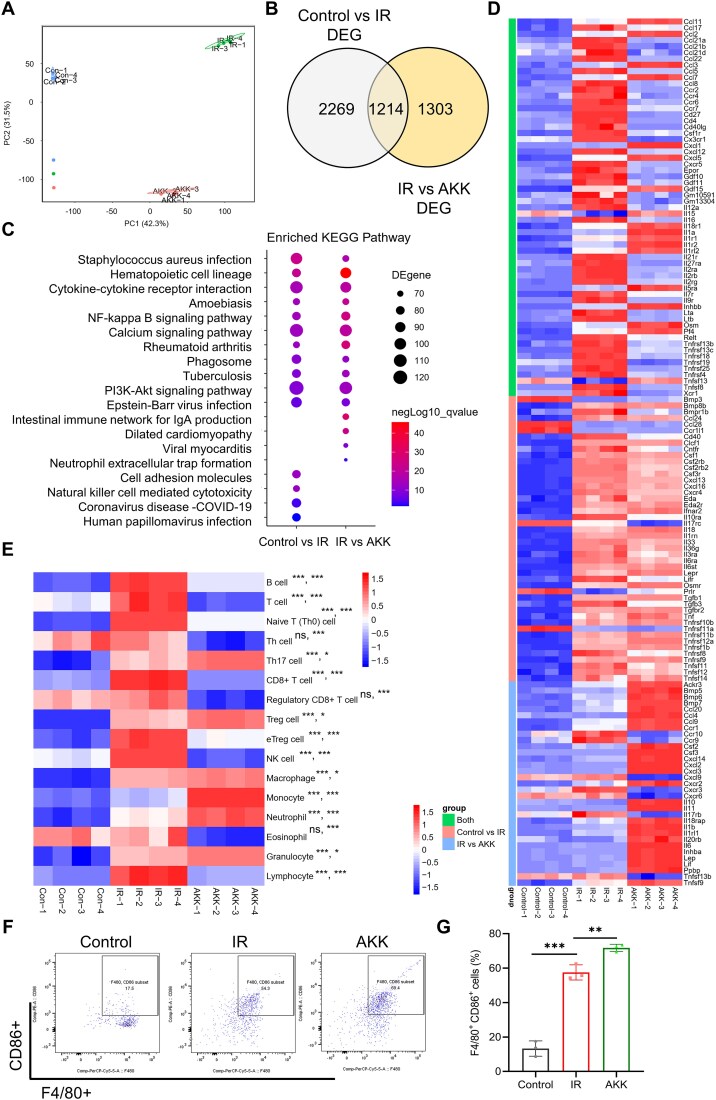
Increased pro-inflammatory Mø in mice following *A. muciniphila* gavage. (A) PCA was performed to assess the alteration of the gene profile from different groups. (B) Venn diagram composed of the differentially expressed genes between control vs. IR and IR vs. AKK groups. (C) KEGG pathway enrichment analysis of DEGs. (D) Heatmap of key genes that are involved in the cytokine–cytokine receptor interaction pathway analysis in the three groups. (E) GSVA analyzed the immune cell infiltration levels. Data scale across rows. The asterisks on the left denote the *P*-value statistics for the control vs. the IR group, whereas the asterisks on the right indicate the *P-value* statistics for the IR vs. the AKK group. (F) The proportion of inflammatory macrophage. ^*^Significance levels: ^*^P < .05, ^*^^*^P < .01.

**Figure 5:**
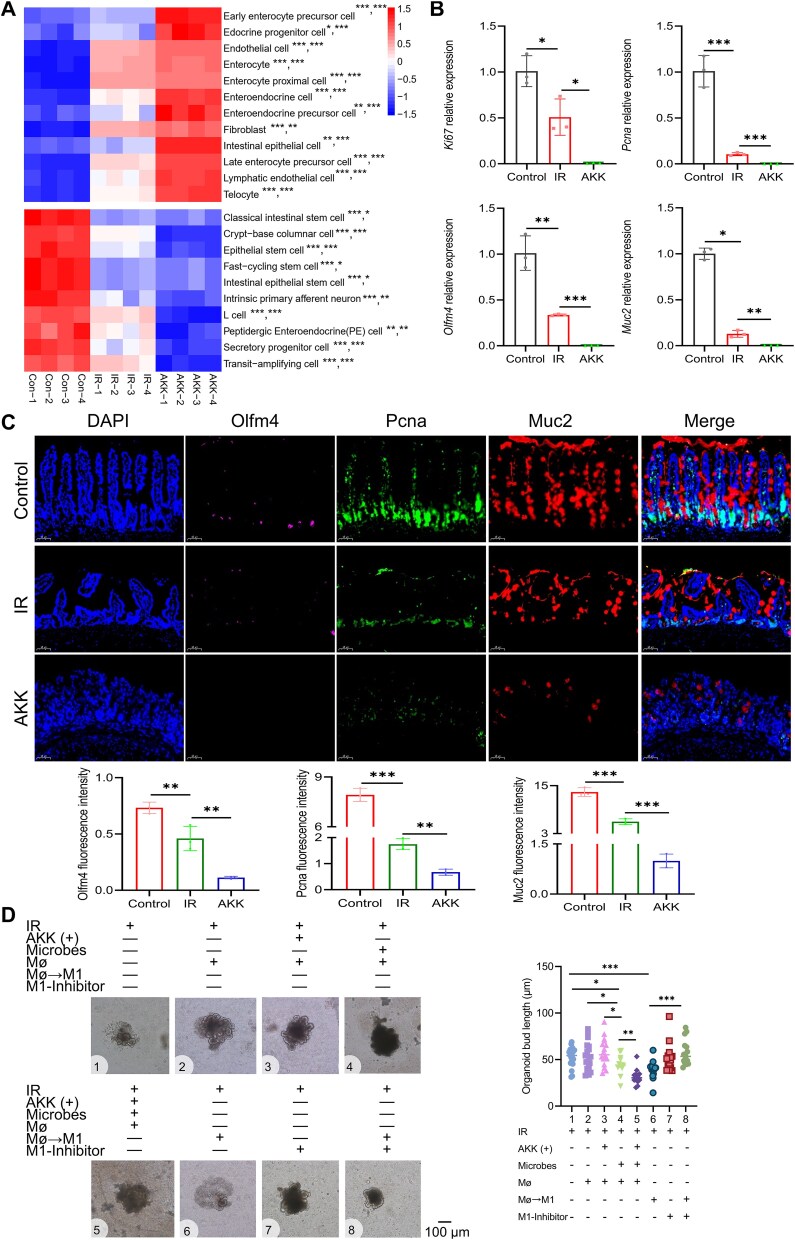
Pro-inflammatory Mø inhibits the intestinal stem cell–TA cell–goblet cell axis. (A) GSVA analysis of intestinal epithelial cell subpopulations. (B) The relative mRNA expression levels of *Ki67, Pcna, Olfm4*, and *Muc2* were examined in jejunum tissues. (C) Multiple immunofluorescence analysis of jejunum and fluorescence intensity quantification. DAPI (blue), Olfm4 (pink), Pcna (green), Muc2 (red), and merged images are shown. (D) Representative images of enteroids cultured under various conditions: IR alone, with/without live AKK (+), with/without *in vitro* gut microbe culture, with/without Mø, with/without M1 Mø, with/without M1 inhibitor. ^*^Significance levels: ^*^  *P* < .05, ^*^^*^P < .01, ^*^^*^^*^P < .001.

### Increased pro-inflammatory Mø in intestine following *A. muciniphila* gavage

To further investigate the effect of AKK on the host, jejunal tissues were collected from each group of mice on Day 4 postirradiation for transcriptome sequencing. PCA confirmed significant differences in the transcriptomic profiles of the control, IR, and AKK groups (Fig. 4A). Differential gene expression analysis identified 3483 genes shared between the control and IR groups and 2517 genes shared between the IR and AKK groups, with an overlap of 1214 genes (Fig. 4B). Kyoto Encyclopedia of Genes and Genomes pathway enrichment analysis of these differentially expressed genes revealed multiple intersecting pathways, including cytokine–cytokine receptor interactions and *Staphylococcus aureus* infection, with the highest gene counts (Fig. 4C). Heatmap analysis of genes within the cytokine–cytokine receptor interaction pathway, such as *IL-1α, IL-6*, and *tumor necrosis factor*, revealed significant changes (Fig. 4D). Immune infiltration analysis using GSVA indicated a sequential increase in Th17 cells, Treg cells, macrophages, monocytes, and neutrophils from the control group to the IR and AKK groups, which correlated with worsening radiation-induced injury (Fig. 4E).

To explore the status of macrophages closely associated with the gut microbiota in mice from each group, we performed flow cytometry and ELISA to detect the numbers of inflammatory macrophages and related cytokines. Flow cytometry revealed that the proportion of inflammatory macrophages in the jejunum was significantly higher in the IR group compared to that in the control group and was even higher in the AKK group (Fig. 4F and G). The results showed progressively elevated levels of the pro-inflammatory factors IL-6 and TNF in the serum and jejunum of mice in the control, IR, and AKK groups, whereas the anti-inflammatory marker transforming growth factor-beta (TGF-β) decreased (Fig. S3A and B). qPCR experiments further demonstrated significant increases in *TNF* concentrations across these groups, whereas the *TGF-β* and *Ccl22* were significantly decreased (Fig. S3C). Collectively, these findings suggest that AKK is associated with increased systemic and local cytokine levels, correlating with intestinal damage.

### Pro-inflammatory Mø inhibits the intestinal stem cell–transit-amplifying cell–goblet cell axis

To explore whether AKK affects intestinal epithelial cells, we conducted a GSVA of intestinal epithelial cell subpopulations using RNA sequencing. The results indicated that there were significantly fewer intestinal stem and TA cells in the AKK group than in the IR group (Fig. 5A). The qPCR results further confirmed significant decreases in the surface markers of stem and TA cells (*Olfm4, Ki67*, and *Pcna*) and the goblet cell marker *Muc2* in the jejunum of the AKK group (Fig. 5B). Multiple immunofluorescence analyses also demonstrated significant reductions in Olfm4, Pcna, and Muc2 expression in the AKK group (Fig. 5C), indicating that AKK suppresses the stem cell–TA cell–goblet cell axis.

To validate the role of the gut microbiota and inflammatory macrophage changes in AKK-mediated suppression of the stem cell–TA cell–goblet cell axis, we performed *ex vivo* validation using enteroids. The results showed that postradiation, addition of macrophages or macrophages + AKK did not significantly worsen ARIII. However, significant exacerbation of injury occurred in the presence of pathogenic gut bacteria or inflammatory macrophage changes, and this damage was partially reversed by treatment with an inhibitor targeting inflammatory macrophages (Fig. 5D). Therefore, AKK exacerbates ARIII dependent on the presence of pathogenic gut bacteria and inflammatory macrophage changes.

### *A. muciniphila* exacerbates ARIII through the presence of pathogenic gut microbiota and inflammatory Mø

**Figure 6:**
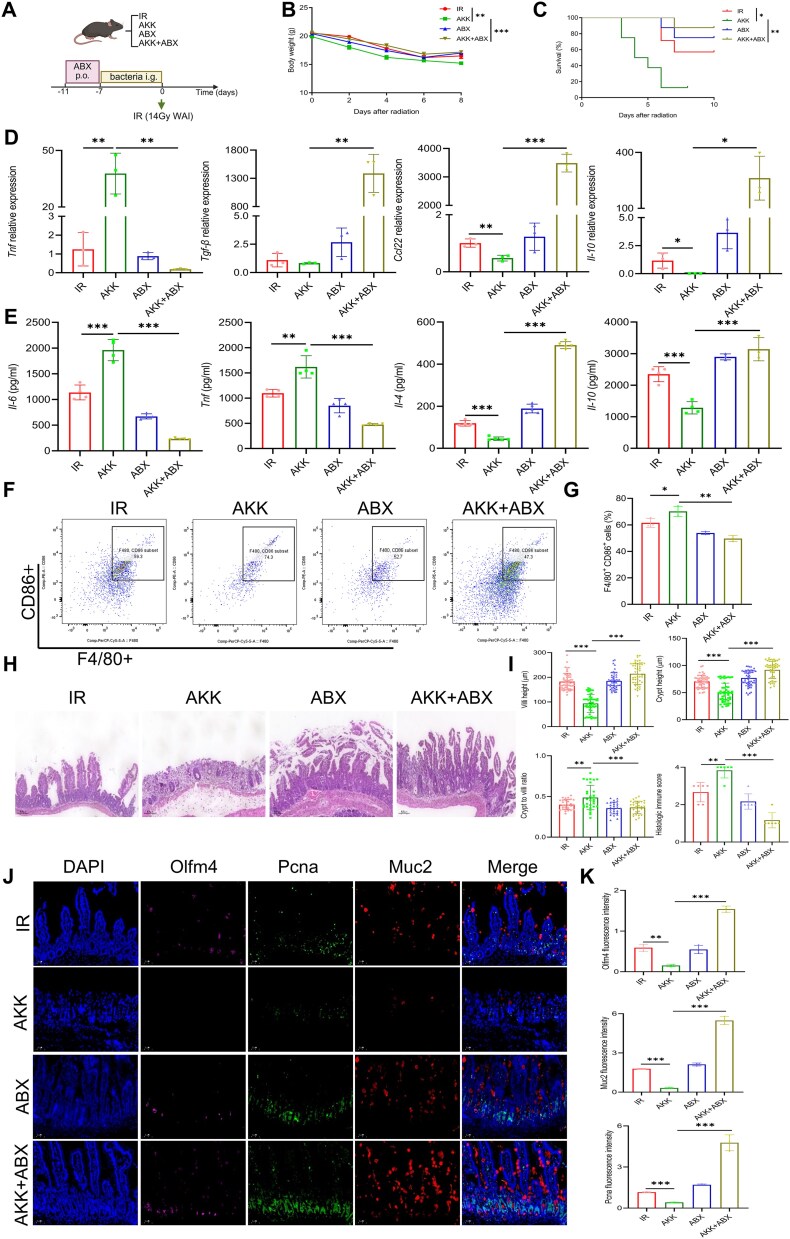
Inhibition of *A. muciniphila* and suppression of pro-inflammatory Mø improve acute radiation-induced intestinal injury. (A) Experimental design of the experiment (IR *n* = 7, AKK *n* = 8, ABX *n* = 8, AKK + ABX *n* = 8). (B) Body weight changes of mice. (C) Survival analysis of mice after postirradiation. (D) The protein levels of *TNF*, *TGF-β*, *Ccl22,* and *IL-10* in the serum of mice. (E) The relative mRNA expression levels of *IL-6*, *TNF*, *IL-4*, and *IL-10* were examined in jejunum tissues. (F) The proportion of inflammatory macrophage. (G) Histological analysis of jejunum sections stained with H&E among different groups. (H) Quantitative assessments of crypt height, villus height, crypt-to-villus ratio, and histologic immune scores. (I) Multiple immunofluorescence analysis of jejunum. DAPI (blue), Olfm4 (pink), Pcna (green), Muc2 (red), and merged images are shown. (J) Fluorescence intensity analysis of Olfm4, Pcna, and Muc2 across the groups. ^*^Significance levels: ^*^P < .05, ^*^^*^P < .01, ^*^^*^^*^P < .001.

We conducted in vivo experiments to confirm that AKK exacerbates ARIII through the presence of pathogenic gut bacteria and inflammatory macrophage changes. Microbiota from mice were decreased with an antibiotic cocktail (Fig. S4A and B), followed by administration of live AKK through oral gavage (Fig. 6A). Analysis of body weight and survival among the groups revealed that mice in the AKK group exhibited lower body weight and survival rates than those in the IR group, a statistically significant difference that disappeared following ABX treatment (Fig. 6B and C). qPCR, ELISA, and flow cytometry demonstrated that AKK no longer significantly promoted inflammatory macrophage changes following ABX treatment (Fig. 6D–G). Histopathological examination of jejunal tissues showed that AKK did not exacerbate ARIII after ABX treatment, as evidenced by the lack of significant differences in villus length, crypt depth, crypt-to-villus ratio, or histological immune score (Fig. 6H and I). Multiplex immunohistochemical analysis indicated that AKK did not inhibit the intestinal stem cell–TA cell–goblet cell axis after ABX treatment (Fig. 6J and K). Thus, AKK exacerbates ARIII, which depends on the presence of other gut bacteria, particularly pathogenic strains.

**Figure 7:**
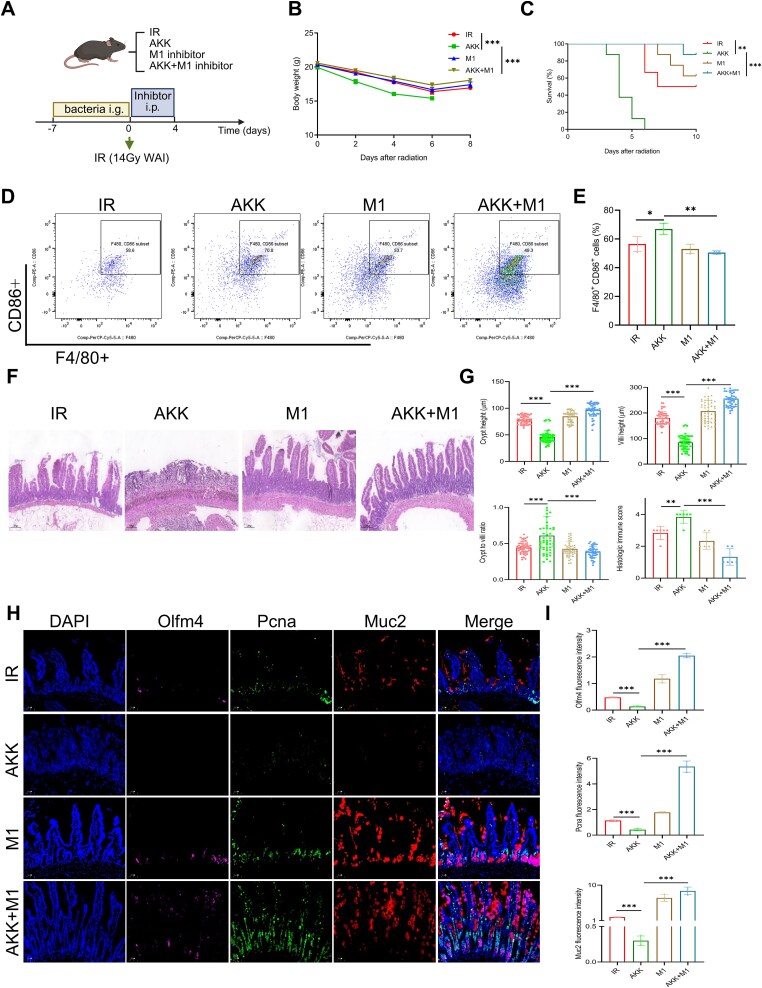
Inhibition of *A. muciniphila* and suppression of pro-inflammatory Mø improve ARIII. (A) Experimental design of the experiment (IR *n* = 6, AKK *n* = 8, M1 *n* = 8, AKK + M1 *n* = 8). (B) Body weight changes of mice. (C) Survival analysis of mice after postirradiation. (D) The proportion of inflammatory macrophage. (E) Histological analysis of jejunum sections stained with H&E among different groups. (F) Quantification of crypt height, villus height, crypt-to-villus ratio, and histologic immune scores. (G) Multiple immunofluorescence analysis of jejunum. DAPI (blue), Olfm4 (pink), Pcna (green), Muc2 (red), and merged images are shown. (H) Fluorescence intensity analysis of Olfm4, Pcna, and Muc2 across the groups. ^*^Significance levels: ^*^P < .05, ^*^^*^P < .01, ^*^^*^^*^P < .001.

To investigate the role of inflammatory macrophage changes in the detrimental effects of AKK, we administered an M1 macrophage inhibitor to mice (Fig. 7A). Evaluation of body weight and survival across the groups revealed that treatment with the M1 macrophage inhibitor improved survival and weight in the AKK group (Fig. 7B and C). Flow cytometry showed that an increased proportion of promoted inflammatory macrophages was rescued by inhibitor treatment (Fig. 7D and E). Histopathological examination of jejunal tissues demonstrated that ARIII exacerbation by AKK was mitigated by inhibitor treatment, with no significant differences in villus length, crypt depth, crypt-to-villus ratio, or histological immune score (Fig. 7F and G). Multiplex immunohistochemical analysis indicated that AKK no longer suppressed the intestinal stem cell–TA cell–goblet cell axis following inhibitor treatment. Therefore, AKK exacerbated ARIII, contingent on the presence of inflammatory macrophage changes (Fig. 7H and I).

## Discussion

In this study, we discovered that a high abundance of AKK in the intestine exacerbated ARIII. Following radiotherapy, the high abundance of AKK in the gut activates mucin metabolism–related genes, facilitating survival by utilizing mucin as a primary energy source. This results in thinning of the mucosal barrier predominantly composed of mucin, thereby promoting adhesion and translocation of potentially pathogenic bacteria, and inducing inflammatory macrophage changes within the intestinal stroma, which secrete pro-inflammatory cytokines such as IL-6 and TNF. This process suppresses the intestinal stem cell–TA cell–goblet cell axis and reduces mucin secretion by goblet cells, thereby establishing a vicious cycle that aggravates ARIII.

Dysbiosis of the gut microbiota plays a crucial role in ARIII [7, 8]. Current clinical trials are gradually adopting microbiota-targeted therapies such as FMT and the diversification of probiotics, although their therapeutic efficacy varies [9, 10, 33, 34]. In this study, we established an ARIII mouse model and performed FMT to explore the role of the gut microbiota in ARIII. Our results demonstrated that abdominal radiation leads to dysbiosis and reduced diversity of the gut microbiota, which is ameliorated by FMT. These findings were consistent with previous studies [35–37].

We observed a significant increase in AKK levels after irradiation, which were effectively reduced by FMT treatment. Supplementation with AKK unexpectedly worsened ARIII and increased mortality, effects that were absent in the pasteurized and heat-inactivated AKK strains. Previous studies have highlighted the important role of AKK in regulating intestinal barrier function [38, 39]. AKK has also been shown to ameliorate ARIII in mice by modulating short-chain fatty acids [40]. Discrepancies in these results may stem from differences in the animal facility or baseline gut microbiota composition (our mice were sourced from southern China, unlike those used in previous studies from northern China), or in the dual role of AKK. Previous studies have extensively documented the adverse effects of AKK on the intestinal environment, particularly under specific conditions such as epithelial barrier damage or during pathogenic infections [25, 26, 41, 42]. Although AKK generally exhibits beneficial effects, its role can be detrimental depending on the environment. Factors such as variations in hosts, intestinal environment, and dietary supplements may influence the abundance and function of AKK, thus potentially exacerbating the risk of pathogenic infections and intestinal inflammation in these pathological contexts [43, 44]. This indicates that addressing dysbiosis related to the pathogenic environment is more relevant than questioning the role of *Akkermansia*.

Previous studies have suggested that AKK can change its probiotic role to a harmful role in certain intestinal environments, thereby exacerbating inflammation. For instance, dietary deficiencies in tryptophan lead to increased AKK colonization, promoting epithelial invasion by pathogens, and exacerbating enteritis [25]. High-sugar and low-fiber diets promote AKK growth, accelerate mucous layer degradation, and worsen colitis [41]. Colonization of AKK in GF IL10^−/−^ mice decreases mucous layer thickness and induces inflammatory cytokines, exacerbating enteritis [26].

After AKK consumes mucin, the mucosal barrier thins, promoting the adhesion and translocation of potential pathogens to epithelial cells and exacerbating intestinal injury [45, 46]. A mouse model of ABX treatment and enteroids suggests that AKK exacerbates intestinal injury depending on the presence of potentially pathogenic bacteria in the gut. These findings are consistent with previous reports highlighting the critical role of the mucosal layer in protecting the intestine from microbial invasion [47]; thinning of the intestinal mucosal layer and increased harmful bacteria may disrupt immune system function, exacerbating inflammatory responses [48]. Loss of mucosal barrier function and reduction in goblet cells make it easier for some symbiotic and pathogenic bacteria to enter the host, enhancing interactions between microbial communities and the intestinal epithelium, thus exacerbating damage to intestinal tissues [46]. Recent studies have confirmed that colonization by Gram-negative rods is a significant factor in severe radiation enteritis [49, 50].

Simultaneously, AKK increases the inflammatory macrophage changes and secretion of inflammatory cytokines during the pathological process of ARIII exacerbation, ultimately suppressing the intestinal stem cell–TA cell–goblet cell axis. Previous studies have established that inflammatory macrophages are key drivers of pro-inflammatory responses in radiation enteritis [13, 16]. Inflammatory macrophages not only directly induce inflammation by secreting pro-inflammatory factors but also attract additional immune cells such as neutrophils and T cells to the site of inflammation, exacerbating local inflammation [51]. Studies have shown that inflammatory intestinal macrophages participate in the physiological activities, repair, and differentiation of intestinal epithelial and stem cells [52]. Research indicates that the loss of macrophages can lead to decreased epithelial stem cell density and altered goblet cell differentiation [14, 53].

**Figure 8:**
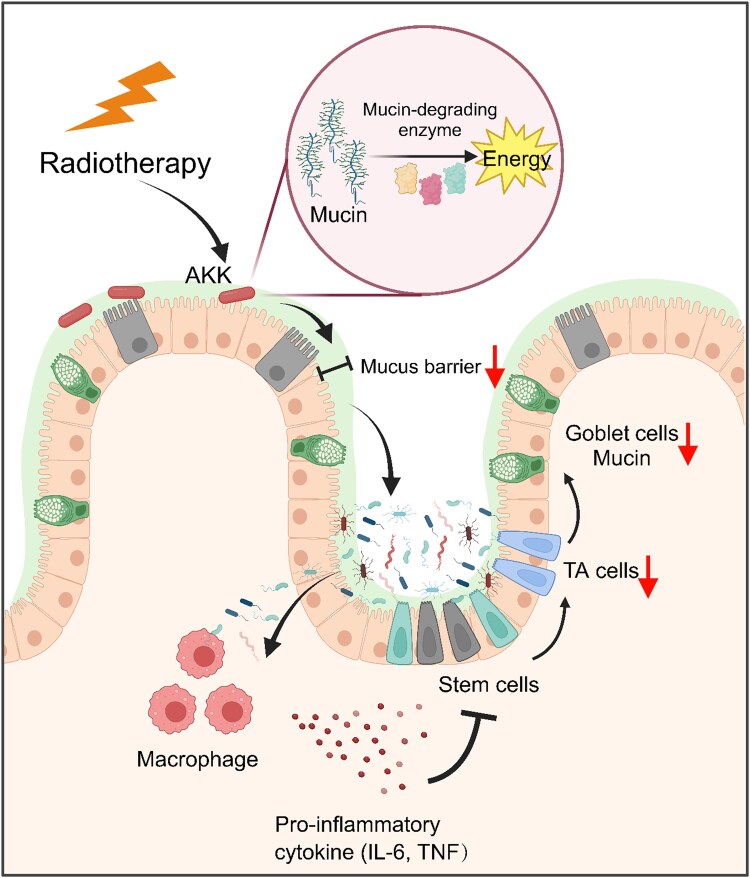
High abundance of AKK in the intestine exacerbates ARIII postirradiation.

We believe that our study makes a significant contribution to the literature because we show that a high abundance of AKK in the intestine exacerbates ARIII. Following radiotherapy, the high abundance of AKK in the gut activates mucin metabolism-related genes, facilitating survival by using mucin as a primary energy source. This results in thinning of the mucosal barrier, predominantly composed of mucin, thereby promoting adhesion and translocation of potentially pathogenic bacteria and inducing inflammatory macrophage changes within the intestinal stroma, which secrete pro-inflammatory cytokines such as IL-6 and TNF. This process suppresses the intestinal stem cell–TA cell–goblet cell axis and reduces mucin secretion by goblet cells, thereby establishing a vicious cycle that aggravates ARIII (Fig. 8).

This study had some limitations. First, we used mice with decreased microbiota through an antibiotic cocktail instead of germ-free mice, and, although ABX significantly reduced the gut bacteria, some intestinal bacteria may have remained. Additionally, we did not extensively study the role of other immune cells, including Th17 cells and Tregs, in the exacerbation of ARIII by AKK. Finally, we exclusively used male mice in this study to avoid hormonal variability and to align with the replacement, reduction, and refinement (3Rs) principles.

The results of this study have significant clinical implications, indicating that although *Akkermansia* exhibits benefits in various animal models [21, 54, 55], such as dextran sulfate sodium (DSS)-induced colitis [24], caution should be exercised before considering its use in the treatment of patients with ARIII.

## Supplementary Material

Figure_S1_wraf084

Figure_S2_wraf084

Figure_S3_wraf084

Figure_S4_wraf084

Supplementary_Table_wraf084

Supplementary_Figures_wraf084

## Data Availability

The metagenomic sequencing data of mouse feces are available in the China National Center for Bioinformation (CNCB) under accession number PRJCA034972 (link: https://ngdc.cncb.ac.cn/bioproject/browse/PRJCA034972). The 16S rRNA gene sequencing data and RNA sequencing data are also available in the same database under accession number PRJCA036431 (link: https://ngdc.cncb.ac.cn/bioproject/browse/PRJCA036431). We confirm that all the data in this manuscript are original, and we have access to the raw data files.
