# Supplementary figures and images for: Akkermansia muciniphila exacerbates acute radiation–induced intestinal injury by depleting mucin and enhancing inflammation
Source: ISME J. 2025 Apr 30;19(1):wraf084. doi: 10.1093/ismejo/wraf084 (PMC12089034; doi:10.1093/ismejo/wraf084)

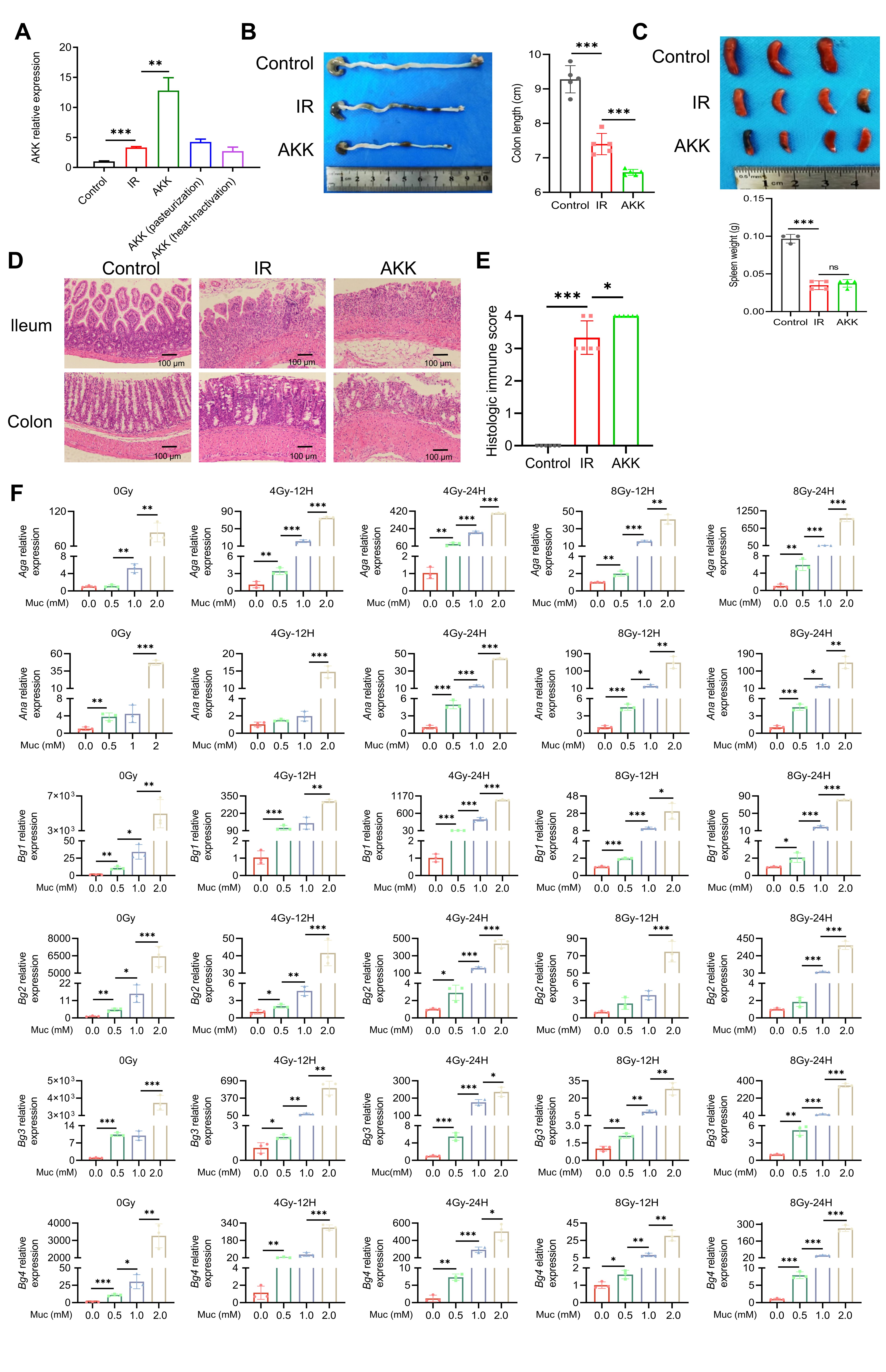

Supplement: Figure_S1_wraf084 [file figure_s1_wraf084.jpeg]

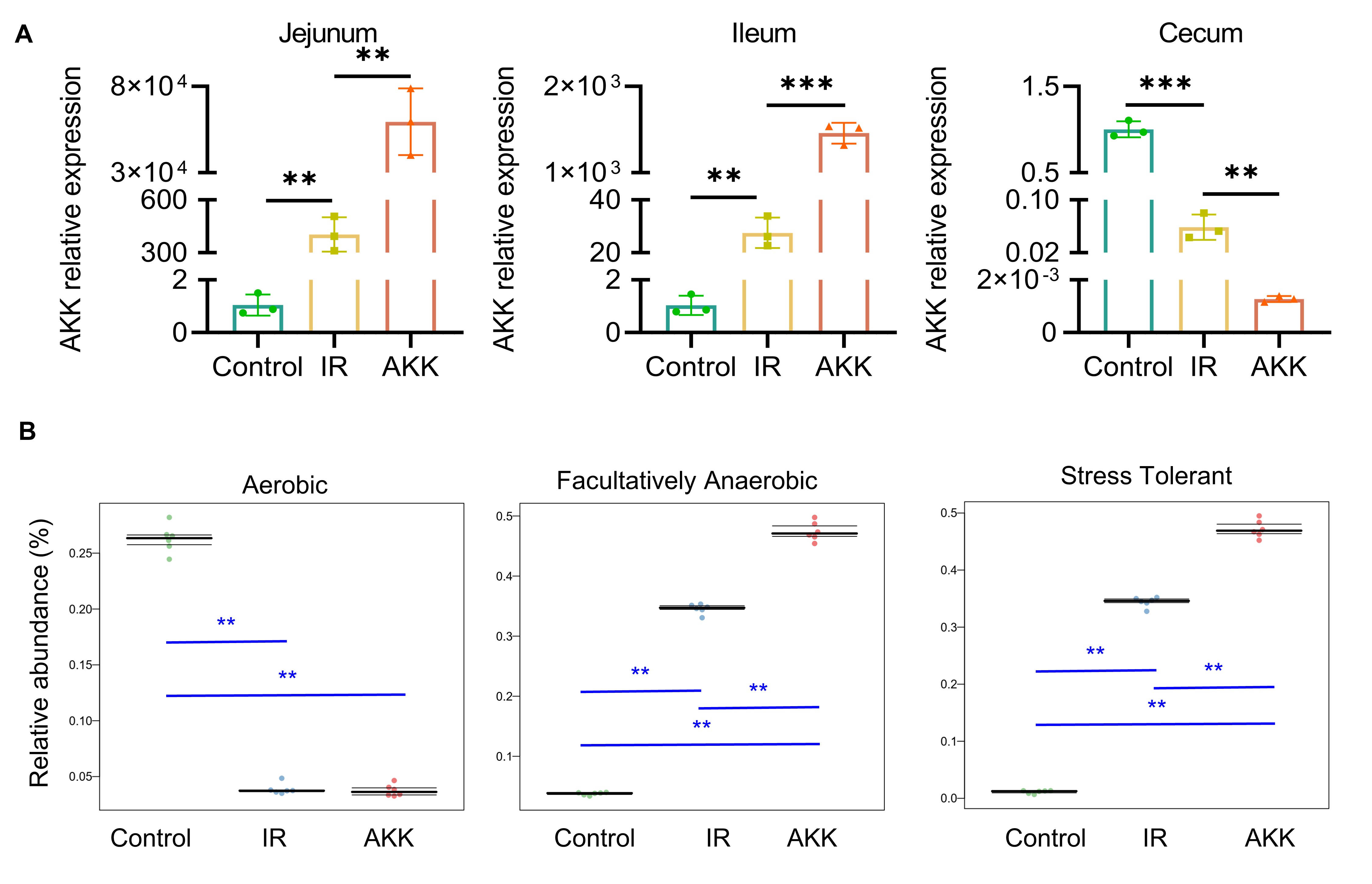

Supplement: Figure_S2_wraf084 [file figure_s2_wraf084.jpeg]

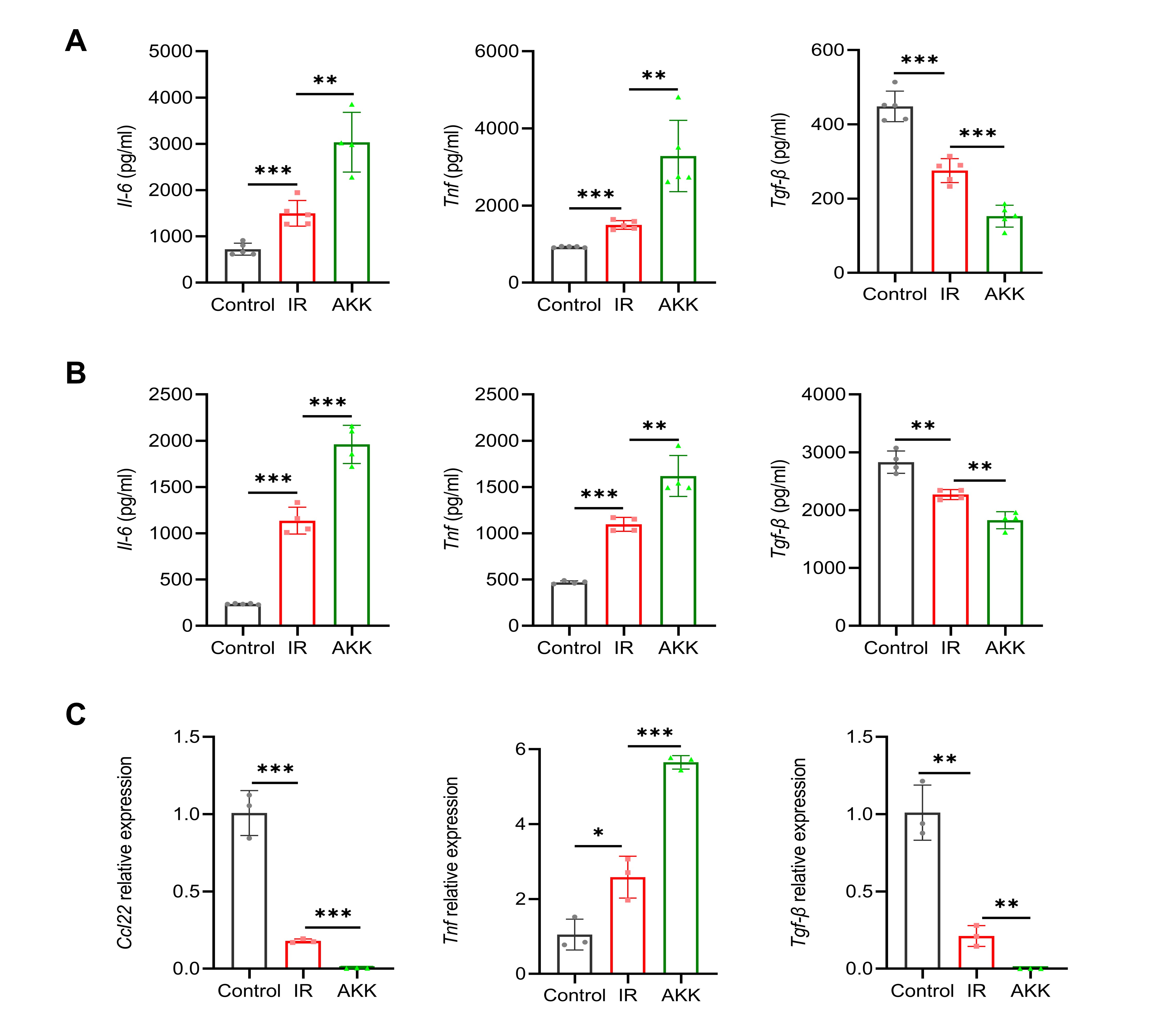

Supplement: Figure_S3_wraf084 [file figure_s3_wraf084.jpeg]

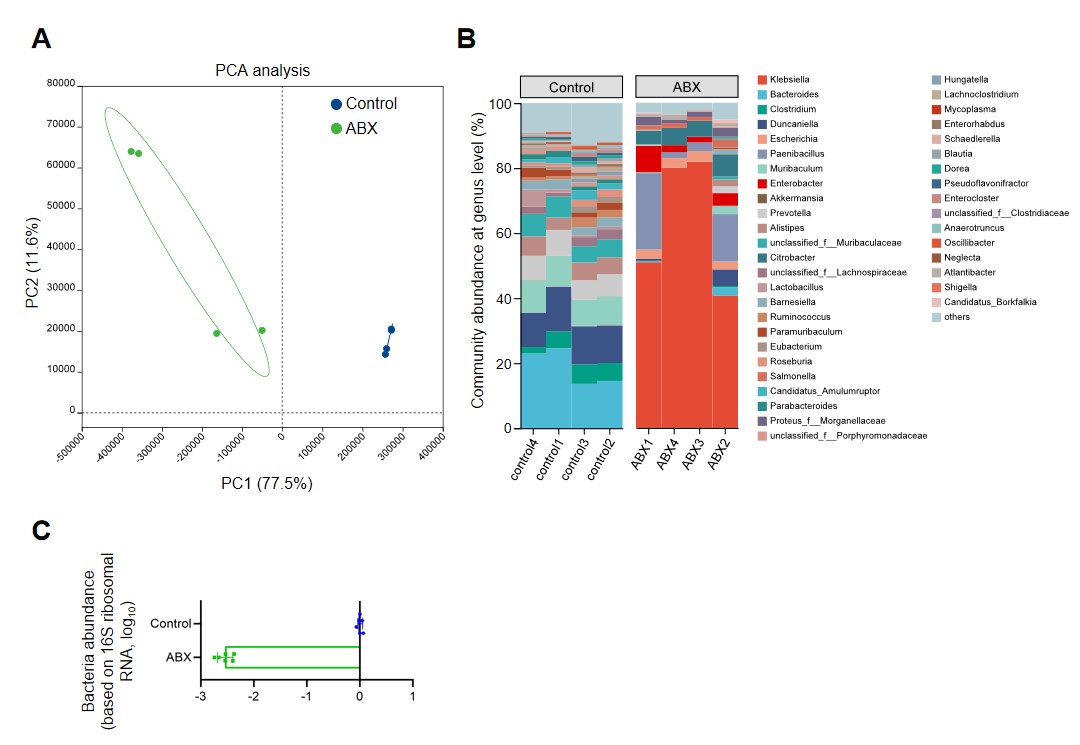

Supplement: Figure_S4_wraf084 [file figure_s4_wraf084.jpeg]
