# Supplementary material for: Akkermansia muciniphila exacerbates acute radiation–induced intestinal injury by depleting mucin and enhancing inflammation
Source: ISME J. 2025 Apr 30;19(1):wraf084. doi: 10.1093/ismejo/wraf084 (PMC12089034; doi:10.1093/ismejo/wraf084)
Supplement: Supplementary_Table_wraf084 [file supplementary_table_wraf084.docx]

**Supplementary Table for**

***Akkermansia muciniphila* Exacerbates Acute Radiation-Induced Intestinal Injury by Depleting Mucin and Enhancing Inflammation**

*Yafang Wang, Xusheng Wang, Zhenhui Chen, Jihua Zheng, Xiangqiang Liu, Yilin Zheng, Zhihao Zheng, Zi Xu, Yaowei Zhang, Keli Chen, Yuqin Zhang*, Lu Yu*, Yi Ding**

**Table S1. Forward and reverse primers of genes.**

**Table S1. Forward and reverse primers of genes.**

| Gene name | Primer | Sequence (5’-3’) |
| --- | --- | --- |
| *AKK*  *16S* | F  R  F | CAGCACGTGAAGGTGGGGAC  CCTTGCGGTTGGCTTCAGAT  GCTCGTGTCGTGAGATGTT |
|  | R | GCTCGTGTCGTGAGATGTT |
| *Alpha-galactosidase, aga* | F | GCCGCTCTGCTCCGCTCAAA |
|  | R | TCGCCTCCCAGCCTTCGTTC |
| *Alpha-N-acetylgalactosaminidase, ana* | F | CCGAAGAAACCAGGAAGCAC |
|  | R | ATATCGCCGCAGGCAAAGT |
| *Beta-galactosidase-1* | F | TTGCGGATCGGGTGTATGA |
|  | R | TGGGAACGAGGTCTTGAGGTAG |
| *Beta-galactosidase-2* | F | CACCAATTTCGGATTCTCGG |
|  | R | CGGCAGTTTCACGCTCTTCT |
| *Beta-galactosidase-3* | F | GGGACGTGACCCTGTTTGC |
|  | R | TGGCGGTTGACACCCTTGA |
| *Beta-galactosidase-4* | F | ACCAGGGTATGCTGATTAACGG |
| *Sialidase* | R  F  R | TTGCTGATGCTCCAGATGAC  CCAGGACCGTGACTGTCGTAT  CGCCGTCATCGCTGTAAAT |
| *GAPDH* | F | TGGCCTTCCGTGTTCCTAC |
|  | R | GAGTTGCTGTTGAAGTCGCA |
| *IL-6* | F | CTGCAAGAGACTTCCATCCAG |
|  | R | AGTGGTATAGACAGGTCTGTTGG |
| *TNF* | F | CCTGTAGCCCACGTCGTAG |
|  | R | GGGAGTAGACAAGGTACAACCC |
| *TGF-β* | F | CTTCAATACGTCAGACATTCGGG |
|  | R | GTAACGCCAGGAATTGTTGCTA |
