# Supplementary material for: Akkermansia muciniphila exacerbates acute radiation–induced intestinal injury by depleting mucin and enhancing inflammation
Source: ISME J. 2025 Apr 30;19(1):wraf084. doi: 10.1093/ismejo/wraf084 (PMC12089034; doi:10.1093/ismejo/wraf084)
Supplement: Supplementary_Figures_wraf084 [file supplementary_figures_wraf084.pdf]

Supplementary Figures for:

Akkermansia muciniphila Exacerbates Acute Radiation-Induced Intestinal Injury by Depleting Mucin and Enhancing Inflammation

Yafang Wang, Xusheng Wang, Zhenhui Chen, Jihua Zheng, Xiangqiang Liu, Yilin Zheng, Zhihao Zheng, Zi Xu, Yaowei Zhang, Keli Chen, Yuqin Zhang, Lu Yu*, Yi Ding**

Figure S1. Characterization of *A. muciniphila* effects on the gastrointestinal tract following radiation exposure.

Figure S2. Phenotypic profiling of gut microbiota in Control, IR, and AKK groups.

Figure S3. Impact of *A. muciniphila* on cytokine levels and macrophage polarization following irradiation.

Figure S4. Impact of antibiotic treatment on gut microbiota composition in Mice.

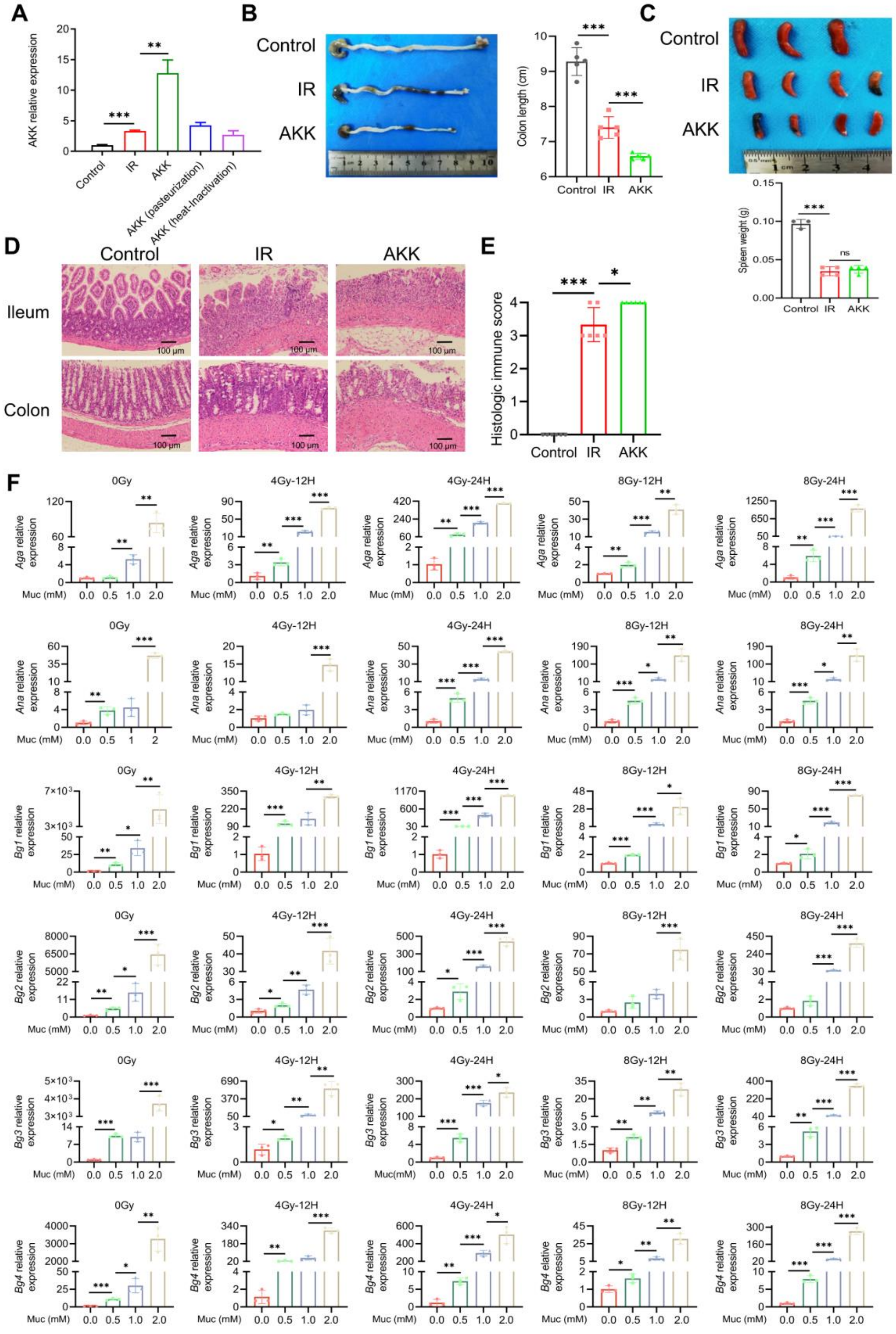

Figure S1. Characterization of *A. muciniphila* effects on the gastrointestinal tract following radiation exposure.

(A) The relative abundance of *A. muciniphila* in the feces was detected by qRT-PCR. (B) Representative photographs of the colons and statistical results of colon length of mice from the control, IR, and AKK groups. (C) Representative images of the spleens and weight across different groups. (D) Histological analysis of ileum and colon sections stained with H&E. (E) Quantitative histologic immune scores of different groups. (F) The relative mRNA expression levels of mucin-utilizing genes (*aga, ana*, *beta-galactosidase*) were examined in *A. muciniphila* post-irradiation. There are four homologous genes of *beta-galactosidase* (*bg1-bg4*) in *A. muciniphila*. *Significance levels: *$P < 0.05$, **$P < 0.01$, ***$P < 0.001$.

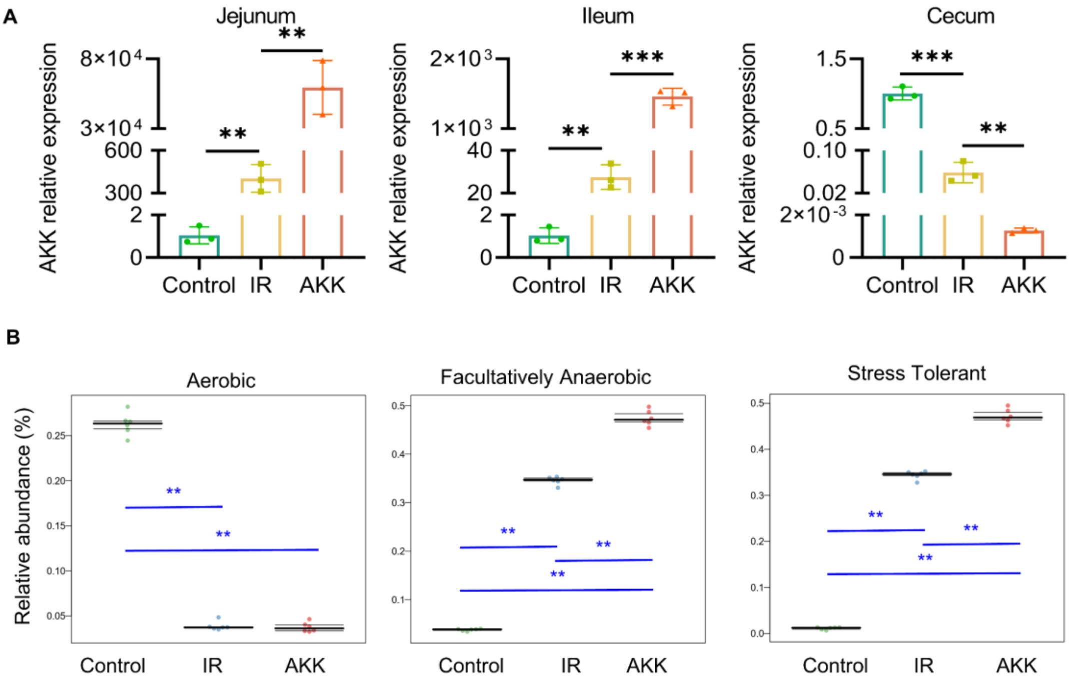

Figure S2. Phenotypic profiling of gut microbiota in Control, IR, and AKK groups. (A) Relative expression of AKK in different intestinal segments. (B) Relative abundance of aerobic, facultatively anaerobic, and stress-tolerant bacterial populations across different groups. *Significance levels: **$P < 0.01$, ***$P < 0.001$.

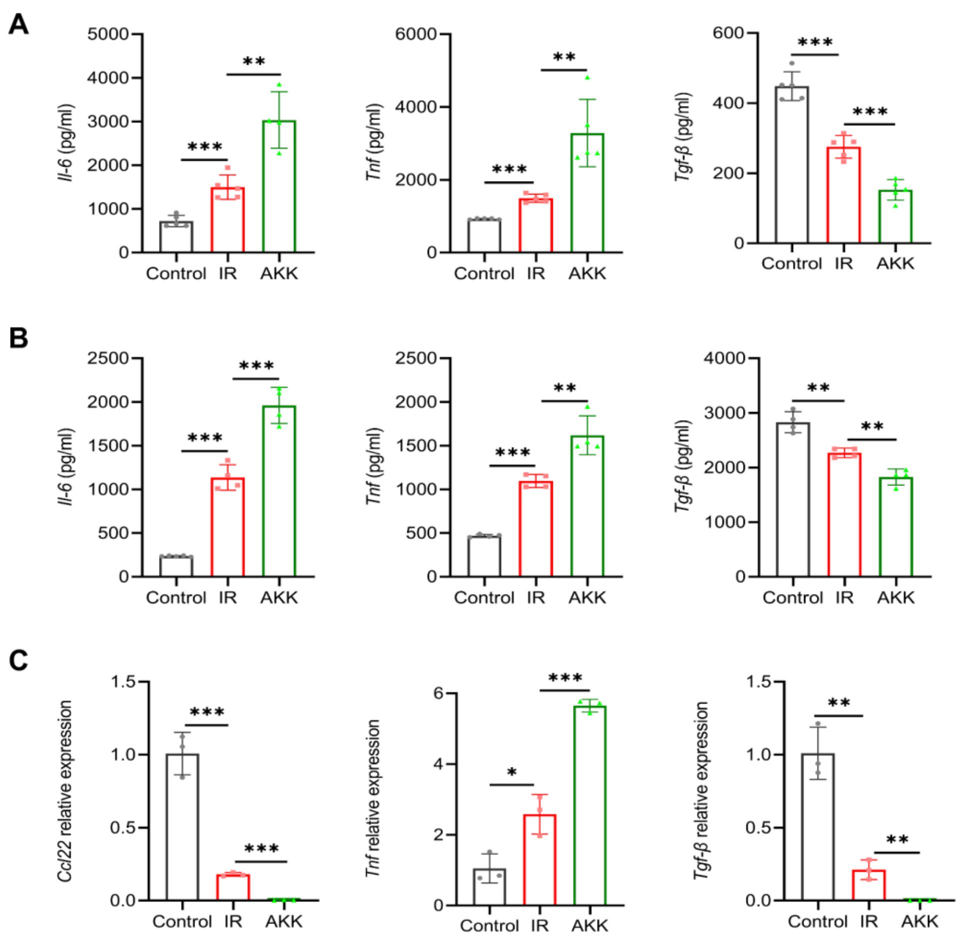

Figure S3. Impact of *A. muciniphila* on cytokine levels and macrophage polarization following irradiation.

(A) The levels of IL-6, TNF, and TGF-β in serum of mice were measured by ELISA across different groups. (B) The levels of IL-6, TNF, and TGF-β in jejunum tissue of mice were measured by ELISA. (C) The relative mRNA expression levels of *Ccl22*, *TNF* and *TGF-β* were examined in jejunum tissues from mice by qRT–PCR. *Significance levels: *$P < 0.05$, **$P < 0.01$, ***$P < 0.001$.

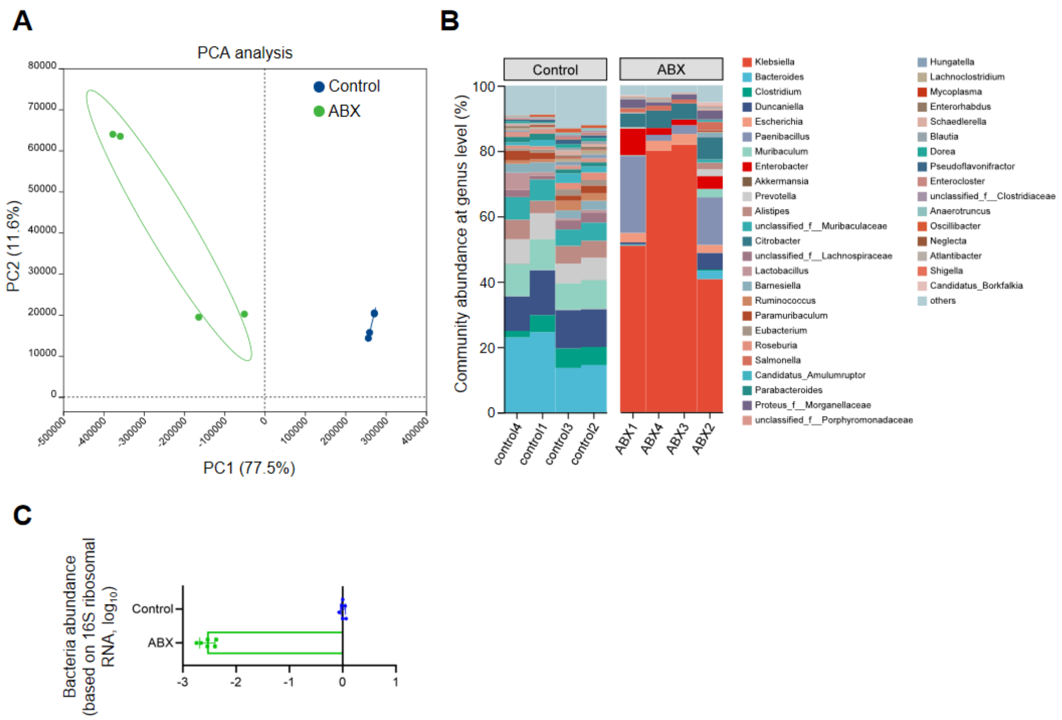

Figure S4. Impact of antibiotic treatment on gut microbiota composition in Mice. (A) PCA of gut microbiome was measured in the three groups. (B) Community barplot at genus level. (C) Abundance of bacteria between Control and ABX treatment groups.
